# The small GTPase Rho5—Yet another player in yeast glucose signaling

**DOI:** 10.1371/journal.pgen.1011858

**Published:** 2025-09-09

**Authors:** Franziska Schweitzer, Linnet Bischof, Stefan Walter, Silke Morris, Hans-Peter Schmitz, Jürgen J. Heinisch

**Affiliations:** 1 Department of Biology/Chemistry, Division of Genetics, University of Osnabrück, Barbarastrasse, Osnabrück, Germany; 2 Department of Biology/Chemistry, Facility for Mass Spectrometry, University of Osnabrück, Barbarastrasse, Osnabrück, Germany; 3 Faculty of Biology, Institute of Integrative Cell Biology and Physiology, University of Münster, Schlossplatz, Germany; Ohio State University, UNITED STATES OF AMERICA

## Abstract

The small GTPase Rho5 has been shown to be involved in regulating the Baker’s yeast response to stress on the cell wall, high medium osmolarity, and reactive oxygen species. These stress conditions trigger a rapid translocation of Rho5 and its dimeric GDP/GTP exchange factor (GEF) to the mitochondrial surface, which was also observed upon glucose starvation. We here show that *rho5* deletions affect carbohydrate metabolism both at the transcriptomic and the proteomic level, in addition to cell wall and mitochondrial composition. Epistasis analyses with deletion mutants in components of the three major yeast glucose signaling pathways indicate a primary role of Rho5 upstream of the Ras2 GTPase in cAMP-mediated protein kinase A signaling. Together with determinations of protein kinase A activities, glycogen and trehalose measurements they indicate a stimulation of Ras/cAMP signaling by Rho5.

## Author summary

GTPases are molecular switches governing a variety of physiological processes and signal transduction cascades in all eukaryotes. Rho5 is a member of the small group of five Rho-type GTPases in the model yeast *Saccharomyces cerevisiae* which modulates several stress response pathways. In this work, the function of Rho5 in yeast glucose signaling has been addressed triggered by the results of proteome and RNA sequence analyses. Different deletion and hyperactive mutant alleles were subjected to extensive epistasis analyses. The observed growth phenotypes, supported by measurements of protein kinase A activity and the accumulation of reserve carbohydrates, indicate an activatory role of the GTPase in the cAMP/Ras signaling pathway, with Rho5 acting upstream of Ras2.

## Introduction

**Fig 1:**
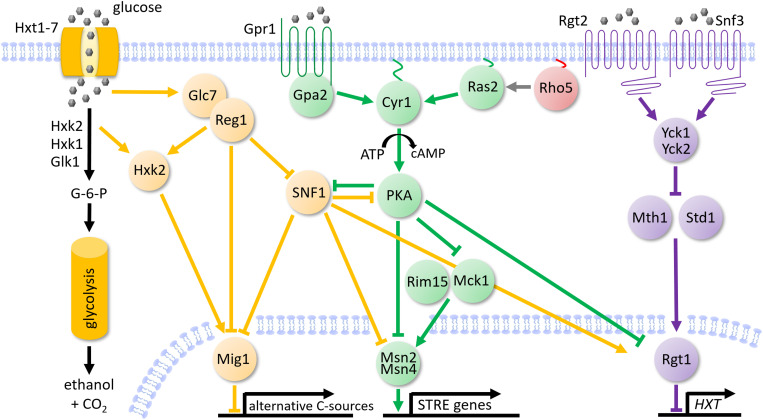
Simplified scheme of glucose signaling pathways in *Saccharomyces cerevisiae.* Glucose (grey pentagons) is internalized by hexose transporters, with a major importance of Hxt1-Hxt7. After activation by hexokinase (primarily Hxk2) it is channeled into glycolysis. At high extracellular glucose concentrations the Reg1-Glc7 phosphatase complex dephosphorylates the SNF1 complex, as well as Hxk2 and Mig1, leading to repression of genes required for the utilization of alternative carbon sources (SNF1 pathway, designated in orange). In the cAMP/PKA pathway (depicted in green) the G-protein coupled receptor Gpr1 senses extracellular glucose and transmits the signal to Gpa2, which activates the adenylate cyclase Cyr1. Cyr1 can also be activated by the redundant Ras1 and Ras2 GTPases in response to intracellular glucose-induced changes. Activated Cyr1 produces cyclic AMP which binds to the regulatory subunits of the heterotetrameric protein kinase A (PKA), triggering its activation. The protein kinases Rim15 and Mck1 are inactivated by PKA-dependent phosphorylation, as are the redundant transcription factors Msn2 and Msn4. A third glucose-responsive pathway is initiated by the Rgt2/Snf3 sensors (shown in violet), which perceive extracellular glucose and activate the yeast casein kinases Yck1 and Yck2. These phosphorylate and thereby mark the cofactors of the transcription factor Rgt1 for proteolytic degradation, namely Mth1 and Std1. The trimeric transcription complex Rgt1/Mth1/Std1 governs the expression of several hexose transporter genes (*HXT*s). Lines ending in bars designate inhibition, arrows indicate activatory functions on target proteins. The proposed role of Rho5 as a positive regulator of Ras2 is also shown.

The yeast *Saccharomyces cerevisiae* has been employed by mankind since thousands of years for making bread and alcoholic beverages like beer and wine [1]. It was thus continuously selected for efficient sugar utilization, with glucose as the preferred carbon source [2]. Besides a large family of hexose transporters [3], this prompted the evolution of complex signaling networks to detect and properly react to the sugar concentration in the medium (see [4–7] for some selected reviews). As outlined in Fig 1, three major signaling pathways have been identified in this context, which are characterized by the trimeric SNF1/AMPK complex, the Rgt2/Snf3 sensor pair, and the cAMP-activated protein kinase A (cAMP-PKA), respectively.

The *SNF1* gene was originally identified in screens for yeast mutants impaired in catabolite repression for utilization of sucrose and other carbon sources than glucose (hence “**s**ucrose **n**on-**f**ermenters”, also designated as *CAT1* [8–10]). It was then cloned and characterized as encoding the protein kinase subunit of a trimeric complex, comprising an additional gamma-subunit (Snf4), and one of three alternative beta-subunits (Gal83, Sip1, Sip2) governing its subcellular localization [11]. Homologues of this trimeric complex in plants and animals, known as AMP-activated protein kinase (AMPK), were also found to govern energy metabolism, with malfunctions having severe effects on human health [12,13]. In yeast, glucose deprivation leads to phosphorylation and activation of the Snf1 kinase subunit by one of three protein kinases (Elm1, Sak1, Tos3), upon which the trimeric complex comprising Gal83 as a ß-subunit enters the nucleus and triggers gene expression for the utilization of alternative carbon sources (see [11] and [7], and references therein, for a detailed overview). Two of its major target proteins are the transcriptional activator Adr1 and the transcriptional repressor Mig1, with the latter being exported from the nucleus upon its phosphorylation. In addition, SNF1 activity exerts a myriad of cellular interactions, which related to this work include the transcription factors Msn2/Msn4, the cAMP-activated protein kinase A (PKA), nutrient signaling through the TORC1 complex, and hexokinase PII [14,15]. Interestingly, Hxk2 also functions as a co-repressor together with Mig1 in nuclear gene expression [16]. Moreover, yeast cell wall synthesis was found to be regulated by the SNF1 complex in a Mig1-dependent manner [17,18].

Glucose signaling mediated by the Snf3/Rgt2 sensors in *S. cerevisiae* acts on the expression of several hexose transporter genes through inactivation of a trimeric repressor complex with Rgt1 as the DNA-binding subunit (Fig 1; reviewed in [4,7]). While transcription of the target *HXT* genes is repressed by limiting glucose concentrations, ample glucose triggers the proteosomal degradation of the Mth1 and Std1 subunits and thereby inactivates the repressor complex. This pathway is crosslinked to SNF1 signaling indirectly by Mig1-mediated repression of genes encoding Rgt1 and its co-repressor Mth1 [19], and directly by phosphorylation and activation of Rgt1 [20].

Finally, Rgt1 repression is also alleviated upon its hyperphosphorylation by protein kinase A (PKA) [21,22], the central component of the third and probably most extensively studied route of yeast glucose signaling (Fig 1; see again [4,7] for general overviews). In brief, glucose signaling in yeast was originally related to the action of the small GTPase homologues of human Ras (Ras1 and Ras2) in stimulating adenylate cyclase [23,24]. Much later, signaling through the G protein coupled receptor (GPCR) Gpr1 mediated by the GTPase Gpa2 was proposed to be the more important trigger of adenylate cyclase activity [25]. In both cases the resulting peak in cAMP concentration leads to dissociation of the inhibitory Bcy1 subunits from the tetrameric PKA complex and concomitant liberation of the catalytic subunits, with the three isoforms Tpk1, Tpk2, and Tpk3 [26,27]. These show partially overlapping but also distinct specificities towards a variety of cytosolic target proteins and nuclear transcription factors (reviewed in [28,29]). Amongst the latter, the redundant transcription factors Msn2/Msn4 are a major target. They are inactivated by phosphorylation and exported from the nucleus in the presence of high glucose concentrations [30,31]. Upon glucose or other nutrient limitations, as well as in response to different environmental stresses (ESR pathway), they reside in the nucleus, where they activate the expression of genes through binding to stress responsive promoter elements (STREs, [32]). In addition to PKA-mediated glucose signaling, nuclear export of Msn2 can also be provoked by its phosphorylation by the protein kinase Rim15, which provides a link to TORC1-mediated nutrient signaling and the regulation of autophagy [33,34].

Despite the fact that this intricate glucose signaling network has been extensively studied in the model yeast *S. cerevisiae* over the past five decades, evidence for new crosstalks is constantly arising. Thus, we found that the small GTPase Rho5 may also take part in nutrient signaling, as *rho5* mutants show strong synthetic defects with *gpa2*, *gpr1*, or *sch9* deletions [35]. Historically, Rho5 was originally identified as a negative regulator of cell wall integrity (CWI) signaling [36], and also found to regulate the opposing high osmolarity glycerol (HOG) pathway [37]. Both pathways are required for proper yeast mitophagy [38]. Moreover, upon exposure to oxidative stress Rho5 rapidly translocates from the plasma membrane to mitochondria, and triggers mitophagy and apoptosis [39,40]. Interestingly, the roles of Rho5 in energy metabolism and mitochondrial functions appear to be conserved in its human homologue Rac1, whose malfunction is associated with several diseases, including diabetes, cancer, and neurodegenerative disorders (reviewed in [41]).

In this work, both the transcriptome and proteome were analyzed in *rho5* mutants and compared to wild-type cells, which substantiated the notion that the small GTPase participates in yeast glucose signaling. We then embarked on more detailed epistasis analyses with deletion mutants in selected components of the three major signaling pathways. Results obtained are consistent with Rho5 acting primarily through the cAMP-PKA pathway.

## Results

### RNAseq and proteome analyses relate Rho5 to glucose signaling and general stress response

**Fig 2:**
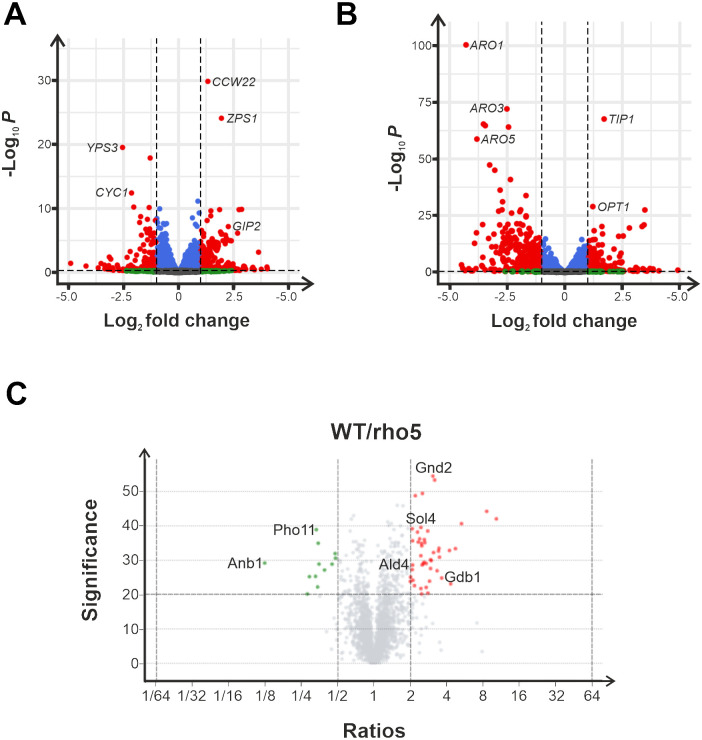
Transcriptome and proteome analyses of *rho5* deletions as compared to wild-type cells. A) Volcano plot of RNAseq data comparing a *rho5* deletion (FSO62-7A) to its isogenic wild-type strain (HD56-5A). Cells were grown on synthetic complete medium with 2% glucose (SCD) and general cut-offs for differentially expressed genes were applied at p-values less than 0.05 and an at least twofold change in transcript abundance, with three biological replicates for each strain. Significant expression changes are depicted in red. Transcripts not meeting the stringent cut-off criteria are designated as follows: Blue colour indicates expression changes with p-values below 0.05 but a fold-change of less than 2. Shown in green are expression changes with a fold-change of at least 2, but a p-value higher than 0.05. Grey transcripts were deemed less significant, as they have p-values higher than 0.05 and a less than twofold change. B) Volcano plot of RNA-sequencing results in which rho5 deletion cells (FSO62-7A) were compared to wild type cells (HD56-5A) after growth on SCD in the presence of 0.8 mM H2O2. Cut-offs and colour codes were applied as in A), again with three biological replicates for each strain. C) Volcano plot of proteins detected in mass spectrometry, comparing a rho5 deletion (FSO62-7A) to wild type cells (HD56-5A) after growth on SCD. Cut-offs were set at p-values less than 0.05 and at least a twofold change in protein abundance. Three biological replicates were used for each strain. Shown in green are all significantly down-regulated proteins, while significantly up-regulated proteins are shown in red. All proteins depicted in grey either have either a p-value higher than 0.05 or a fold-change of less than 2.

As Rho5 has been found to be involved in the regulation of a large number of signaling processes [42], we decided to apply two global assays to assess the effect of *rho5* deletion mutants on yeast physiology. First, data on the transcriptome were obtained by RNAseq for the wild-type and the deletion mutant under standard growth conditions in synthetic medium with 2% glucose, both in the absence and presence of 0.8 mM hydrogen peroxide. A total of 99 genes showed significant upregulation in their expression under standard growth conditions when the deletion mutant was compared to the wild-type, whereas 95 genes appeared to be downregulated (Fig 2A; cut-offs applied at p-values less than 0.05 and at least a twofold change in expression; see S1 Table for a complete list of up- and downregulated genes).

As would be expected from the established Rho5 functions, the upregulated genes included those encoding cell wall and mitochondrial proteins (Table 1). In addition, a number of genes related to carbohydrate metabolism were upregulated, including several hexose transporter genes (*HXT*s) and the glucose-repressed genes *HXK1* and *GLK1*, which encode hexokinase I and glucokinase, required for sugar consumption in the late phase of wine fermentations. Moreover, genes encoding key enzymes of the pentose phosphate pathway, stress protection and reserve carbohydrate metabolism were found to be upregulated in the *rho5* deletion as compared to the wild type. Of note, many of these genes and those placed into the other metabolic groups carry stress-responsive elements (STREs) in their promoters (Table 1).

**Table 1:** Selected genes/proteins differentially expressed in a *rho5* deletion as compared to wild type.

Gene name	Protein function	RNA Seq [fold change]	RNA Seq [p-value]	Mass Spec [fold change]	Mass Spec [p-value]	STRE^b^
Glucose uptake and activation						
*HXK1*	Hexokinase isoenzyme 1	1.05	1.10 x 10^–2^	1.57	1.80 x 10^–3^	Yes
*HXT11*	Hexose transporter	-6.65	1.96 x 10^–6^	nd	nd	
*HXT13/15/16/17* ^*a*^	Putative transmembrane polyol transporter	nrc	nrc	1.18	4.91 x 10^–2^	
*HXT5*	Hexose transporter with moderate glucose affinity	2.33	1.03 x 10^–5^	nd	nd	Yes
*HXT6* ^*a*^	High-affinity glucose transporter	1.95	1.05 x 10^–6^	3.57	1.05 x 10^–3^	
*HXT7* ^*a*^	High-affinity glucose transporter	2.75	1.53 x 10^–10^	3.57	1.05 x 10^–3^	
Pentose Phosphate Pathway						
*GND2*	6-phosphogluconate dehydrogenase	1.74	3.70 x 10^–4^	1.63	3.68 x 10^–6^	
*NQM1*	Transaldolase of unknown function	1.20	4.70 x 10–3	1.79	4.60 x 10^–4^	Yes
*SOL4*	6-phosphoglucono lactonase	1.47	7.18 x 10^–5^	1.06	1.10 x 10^–4^	
*TKL2*	Transketolase	2.69	7.35 x 10^–7^	1.39	1.19 x 10^–3^	Yes
Stress protection and reserve carbohydrates						
*GDB1*	Glycogen debranching enzyme (degradation)	1.36	7.52 x 10^–3^	1.87	3.40 x 10^–3^	
*GLC3*	Glycogen branching enzyme (accumulation)	1.53	5.33 x 10^–6^	nrc	nrc	
*GPH1*	Glycogen phosphorylase (mobilization)	1.81	7.79 x 10^–5^	1.65	6.20 x 10^–4^	Yes
*GSY1*	Glycogen synthase isoenzyme 1	1.62	6.68 x 10^–5^	1.43	4.65 x 10^–2^	
*GSY2*	Glycogen synthase isoenzyme 2	1.09	1.62 x 10^–3^	1.06	1.30 x 10^–4^	Yes
*PGM2*	Phosphoglucomutase	1.35	1.35 x 10^–3^	1.34	1.94 x 10^–2^	Yes
*TFS1*	Inhibitor of Ras GAP (Ira2p) and carboxy-peptidase Y (Prc1p)	1.40	5.05 x 10^–5^	1.58	1.06 x 10^–3^	Yes
*TPS2*	Phosphatase subunit of T-6-P synthase/phosphatase	1.27	3.00 x 10^–4^	1.32	3.90 x 10^–4^	Yes
*TSL1*	Large subunit of the T-6-P synthase/phosphatase	1.31	1.36 x 10^–3^	1.41	3.20 x 10^–4^	Yes
Cell surface and cell wall architecture						
*ECM4*	S-glutathionyl-(chloro) hydroquinone reductase	1.02	8.96 x 10^–5^	1.32	9.89 x 10^–3^	Yes
*EIS1*	Eisosome component required for assembly	nrc	nrc	1.01	1.93 x 10^–2^	Yes
*LSP1*	Eisosome core component	nrc	nrc	1.20	1.60 x 10^–4^	Yes
*PIR3*	Cell wall protein	-1.41	8.02 x 10^–6^	nd	nd	
*SCW10*	Cell wall protein	-1.15	2.61 x 10^–3^	nrc	nrc	
*YLR042C*	Cell wall protein	-1.91	1.88 x 10^–3^	nd	nd	
Mitochondrial functions						
*ALD4*	Aldehyde dehydrogenase	nrc	nrc	1.07	1.42 x 10^–3^	Yes
*CYB5*	Cytochrome b5	-1.13	7.13 x 10^–4^	nrc	nrc	
*CYC1*	Cytochrome c, isoform 1	-2.14	3.78 x 10^–13^	nrc	nrc	
*GUT2*	Glycerol-3-phosphate dehydrogenase	ns	ns	1.14	1.36 x 10^–5^	
*NCA3*	Protein involved in mitochondrion organization	-1.55	5.10 x 10^–4^	nd	nd	
*NDE2*	External NADH dehydrogenase	1.16	1.89 x 10^–2^	nd	nd	
*OM14*	Mitochondrial outer membrane receptor for cytosolic ribosomes	ns	ns	1.06	1.94 x 10^–3^	
*OM45*	Mitochondrial outer membrane protein of unknown function	1.56	8.08 x 10^–5^	1.78	5.60 x 10^–4^	
*PIC2*	Copper/phosphate carrier	1.14	2.44 x 10^–6^	nd	nd	
*SFC1*	Succinate-fumarate transporter	1.07	2.66 x 10^–2^	nd	nd	
*YMC2*	Putative mitochondrial inner membrane transporter	-1.12	2.37 x 10–3	nrc	nrc	

nrc = no relevant changes; nd = not detected.

^a^Due to high sequence similarity, these yeast hexose transporters cannot be differentiated from the peptides detected in mass spectrometry. Data could thus reflect the concentration changes in either one of these transporters or any combination of them.

^b^Where stress responsive elements (STREs) have either been shown to function in gene expression, or at least were detected in bioinformatic surveys [43], their presence is indicated as “yes”.

Under oxidative stress, i.e., exposure to 0.8 mM H_2_O_2_ for six hours, candidate genes involved in the general or environmental stress response (ESR) were upregulated both in the wild-type and the *rho5* deletion strains (Fig 2B and S2 Table). Differential regulation between the two genetic backgrounds was observed for 120 genes (upregulation) and for 276 genes (downregulation; S2 Table).

In order to confirm the validity of the RNAseq results, expression of a subset of genes from the different groups listed in Table 1 was investigated by real-time RT-PCR (*CCW22, CYC1, ECM4, GPH1, GSY2, HXK1, HXT6/7, OM45, PGM2, TPS2*; S1 Fig). Comparison between wild-type and the *rho5* deletion grown under standard conditions confirmed the increase in expression levels for all genes upregulated in RNAseq. We attribute differences in the exact fold-changes detected by the two methods to the relatively moderate increases in gene expressions observed, and to the error margins associated with the real-time RT-PCR method, e.g., in determining the exact concentration of the template cDNA. In contrast to what was observed in RNAseq, *CYC1* expression increased in the RT-qPCR on the *rho5* deletion as compared to the wild-type. Besides the error margins just mentioned, this could be explained by the overall low expression of the gene, with minor changes in growth conditions leading to strong effects on its representation in the cDNA pool.

**Fig 3:**
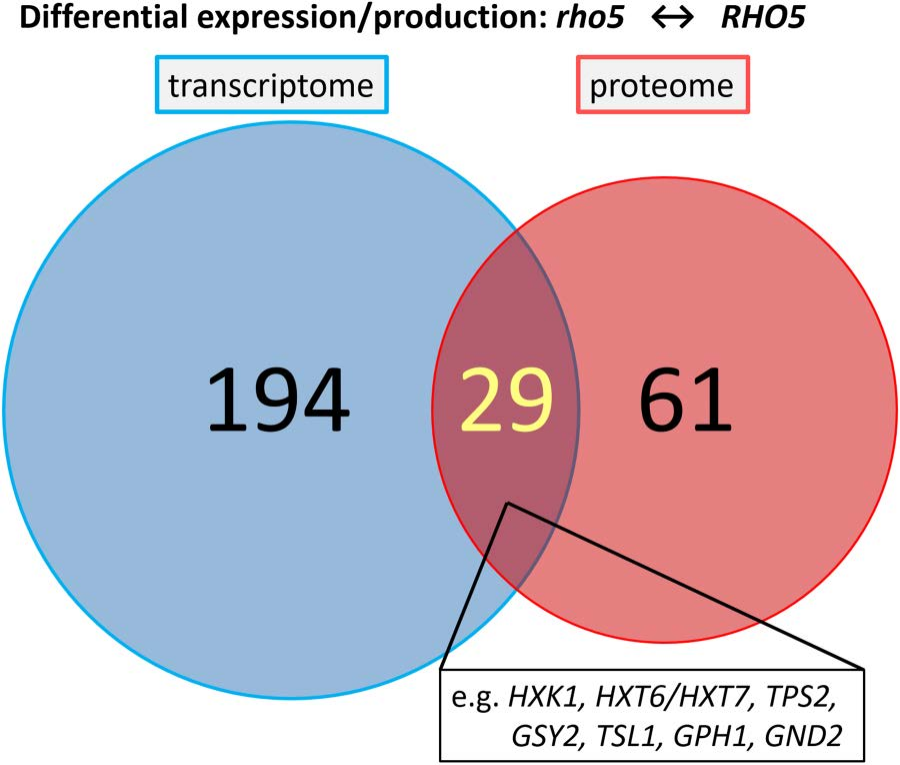
Venn-Diagram of overlapping data obtained from RNA-sequencing and mass spectrometry from comparison of a *rho5* deletion (FSO62-7A) to a wild-type strain (HD56-5A) after growth on SCD. Cut-off criteria for significant differences were the same as described in the legend of Fig 2.

In a complementary approach to the gene expression analyses, differential protein concentrations were assessed for the same strains grown under standard growth conditions using mass spectrometry. A total of 64 proteins increased significantly in their amount when the deletion mutant was compared to the wild type, whereas only 14 proteins showed a decreased concentration (Fig 2C; with cut-offs applied at p-values less than 0.05 and at least a twofold change in protein abundance; see S3 Table for a complete list of affected proteins). Again, proteins most strongly affected by the *rho5* deletion included those involved in carbohydrate metabolism (Table 1), with a number of them overlapping with and confirming the RNAseq data (Fig 3).

### Epistasis analyses reveal genetic interactions of *RHO5* with glucose signaling through the SNF1 complex and hexokinase

The global expression data suggested a relationship between Rho5 and carbohydrate metabolism. We therefore proceeded by assessing the phenotypes of either a *rho5* deletion or the hyper-active *RHO5*^*G12V*^ allele in combination with different mutants in the major glucose signaling pathways. For this purpose, classical genetic crosses were performed, the resulting diploids were subjected to tetrad analyses, and growth was first monitored by determination of colony sizes of the different mutant and wild-type segregants on rich medium plates with 2% glucose as a carbon source.

**Fig 4:**
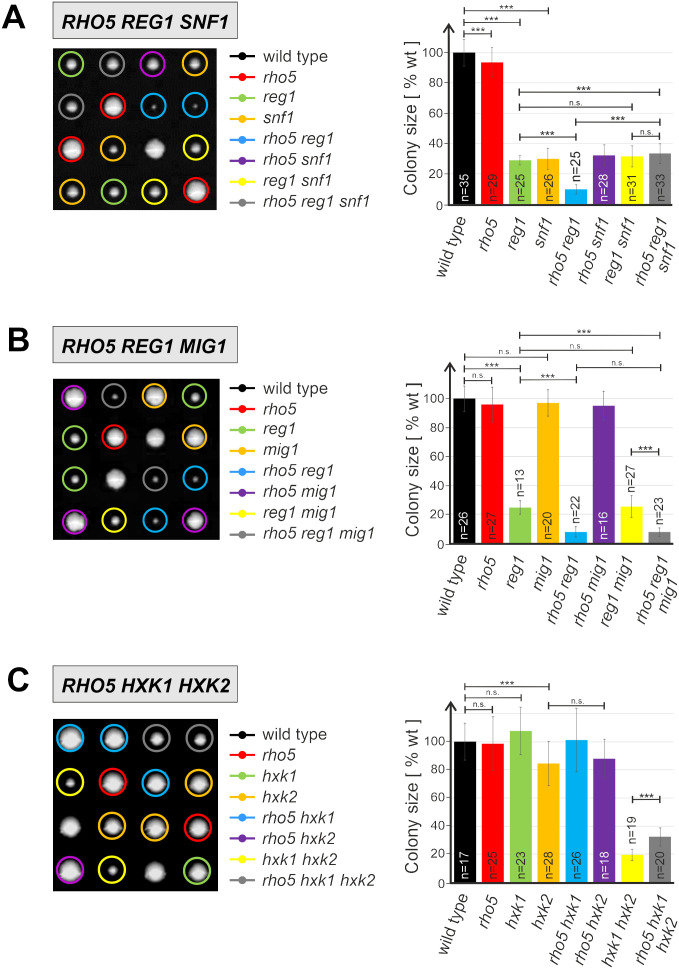
Epistasis analyses based on growth of segregants from tetrad analyses on rich medium (YEPD). Plates were incubated for three to five days at 28°C, depending on the crosses to be analyzed. Only four exemplary tetrads are shown for each cross, with colored circles designating different combinations of gene deletions as indicated. Colony sizes for each combination (determined from pixel area and given as percentage from wild type set at 100%) were determined from at least 40 tetrads from each cross and quantified in the columns of the diagram at the right (n = total number of segregants obtained for each genotype; error bars are indicated for each data set, three asterisks indicate highly significant differences with p-values below 0.001; n.s. = not significant). Diploids analyzed were obtained from the following crosses: A) A strain carrying a rho5 snf1 double deletion (FSO67-2C) with a reg1 snf1 double deletion (FSO66-3C). B) A strain carrying a rho5 deletion (FSO71-2A) with a reg1 mig1 double deletion strain (FSO79-8C). C) A strain carrying a rho5 deletion (FSO43-1D) with one carrying a hxk1 hxk2 double deletion (HOD257-2B).

As shown in Fig 4A, segregants with a *rho5* deletion form slightly smaller colonies than those with the wild-type allele. However, the growth area is reduced by approximately threefold in segregants lacking either the kinase subunit of the SNF1 complex (*snf1Δ*) or the Reg1 subunit of its phosphatase (*reg1Δ*), which is required for its inactivation (note that in lack of a hyper-active Snf1 kinase derivative *reg1* deletions are commonly employed to constitutively activate the SNF1 complex [44]). Interestingly, an additional *rho5* deletion aggravates the growth defect of strains lacking Reg1, while it does not alter growth of the *snf1* deletions (Fig 4A). The fact that the slow growth of *rho5 reg1* strains is restored back to that of a single *reg1* deletion by an additional lack of Snf1, i.e., in *rho5 reg1 snf1* triple deletions, suggests that Rho5 negatively affects the activity of the SNF1 complex. These growth impairments derived from colony sizes were also confirmed by recording growth curves in liquid synthetic medium, suggesting that they are indeed owed to differential growth rates, rather than a delay in spore germination (S2A Fig).

As the transcription factor Mig1 is a major downstream target of SNF1 signaling in carbohydrate metabolism, epistatic relationships were also investigated with a *mig1* deletion. Surprisingly, the growth retardation observed in a *rho5 reg1* double deletion, amounting to less than 10% of wild-type segregants, was not alleviated in a triple *rho5 reg1 mig1* deletion (Figs 4B and S2B), demonstrating that the observed Rho5- and Snf1-dependent growth effects are not mediated by Mig1. By contrast, the growth defect caused by a lack of Snf1 requires a functional Mig1, as it is relieved in strains with a *snf1 mig1* double deletion (S3 Fig).

In parallel to the SNF1 complex, hexokinase PII was among the first components found to participate in yeast glucose repression [45,46]. Besides its association with Mig1 in the nucleus at high external glucose concentrations, its inhibitory action on the SNF1 complex is probably associated with its catalytic activity, but still somewhat enigmatic (reviewed in [47]). *HXK2* encodes one of three yeast isozymes capable of glucose phosphorylation, together with a glucokinase encoded by *GLK1* and another hexokinase encoded by *HXK1*. Expression of the latter two is subject to glucose repression and only *hxk1 hxk2 glk1* triple deletions cannot grow on glucose as a sole carbon source [48,49]. As expected, we found only a moderate decrease in colony sizes after tetrad analyses for *hxk2* deletions compared to wild-type segregants, whereas those of *hxk1 hxk2* double deletions were reduced by approximately 80% (Fig 4C). Interestingly, this phenotype could be partially alleviated by an additional *rho5* deletion, which restored growth of the triple mutants to approximately 30% of that of the wild-type colonies. Again, these findings were substantiated by recording growth curves of segregants with the different mutant combinations (S2C Fig).

We attribute the slight positive effect of the *rho5* deletions to an increase in respiratory capacity (S4 Fig), which may counteract the reduced energy supply caused by the *hxk1 hxk2* deletions. In this case, the hyper-active Rho5^G12V^ variant also caused a minor, though less significant increase in respiration, instead of the expected decrease, indicating that lack of the GTPase affects energy metabolism more strongly than its stimulation.

The slow growth phenotype of the *reg1* deletion aggravated by the additional lack of Rho5 described above appeared to be intriguing. As demonstrated in the following section of results, we found evidence for Rho5 acting upstream of the Ras2-GTPase in cAMP signaling (see also Fig 1). Therefore, colony sizes were also determined in epistasis analyses involving mutant alleles of these three genes. As evident from S5A Fig, a lack of Ras2 also aggravates the phenotype of a *reg1* deletion similar to the *rho5 reg1* double deletion. *Vice versa*, introduction of the hyper-active *RAS2*^*G19V*^ allele suppresses the slow growth defect of *rho5 reg1* segregants (S5B Fig; note that *RAS2*^*G19V*^ prevents sporulation of yeast diploids through its inhibitory action on the master transcriptional regulator Ime1. Therefore, we overexpressed *IME1* in the respective diploids by introducing a high-copy number plasmid with the gene under the control of the strong yeast *PFK2* promoter to circumvent the problem, restore ascus formation and allow tetrad analysis).

### *RHO5* genetically interacts with cAMP-PKA signaling

**Fig 5:**
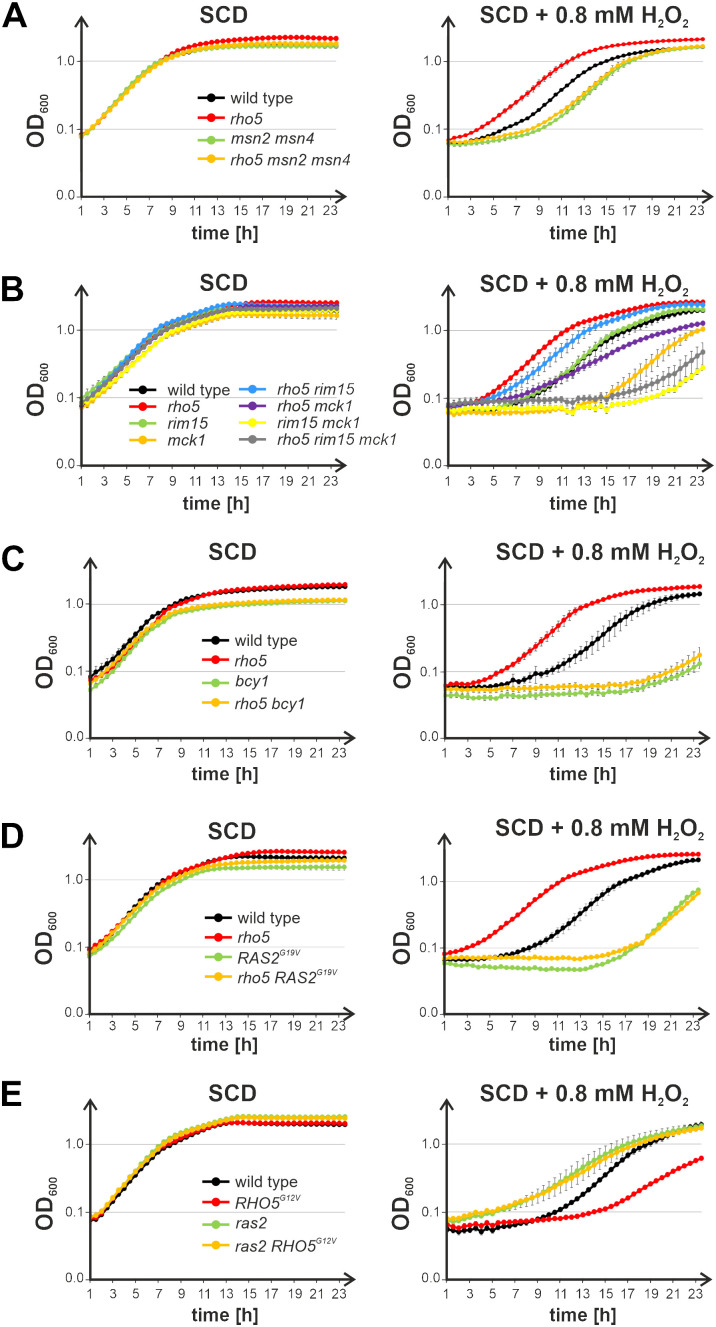
Epistasis analyses based on the sensitivity towards hydrogen peroxide of strains carrying mutations in genes encoding components of the cAMP-PKA signaling pathway in combination with *RHO5* variants. Growth curves were recorded on synthetic medium with 2% glucose (SCD; supplemented with 4 mg/L histidine for strains with a histidine auxotrophy), in the absence and presence of hydrogen peroxide as indicated. Error bars give the standard deviations at each time point obtained from at least two biological and two technical replicates from parallel measurements for each curve (i.e., two independent isogenic segregants were measured, with two independent inoculates, each). Genotypes of the otherwise isogenic strains are listed in Table 2. Strains employed were A) wild type (FSO36-5B and FSO36-11A), *rho5* (FSO36-11D and FSO36-5D), *msn2/4* (FSO36-11B and FSO36-5A) *rho5 msn2/4* (FSO36-5C and FSO36-11C). B) wild type (FSO86-7A and FSO86-2A) *rho5* (FSO105-7D and FSO105-1C) *rim15* (FSO86-6B and FSO86-1C) *mck1* (HOD343-2A and HOD343-4A) *rho5 rim15* (FSO86-7C and FSO86-2C) *rho5 mck1* (HOD343-2C and HOD343-4C) *rim15 mck1* (FSO84-4A and FSO84-10B) *rho5 rim15 mck1* (FSO84-10D and FSO84-3D). C) wild type (FSO16-3B and FSO86-7A) *rho5* (FSO105-7D and FSO105-1C) *bcy1* (FSO105-4A and FSO105-1B) *rho5 bcy1* (FSO105-1D and FSO105-7C). D) wild type (FSO35-4A and FSO36-5B) a *rho5* deletion strain (FSO36-11D and FSO105-7D), a *RAS2*^*G19V*^ mutant strain (HOD610-1D/ RAS2^G19V^URA and HOD610-2C/ RAS2^G19V^URA) and a *rho5 RAS2*^*G19V*^ mutant strain (HODrk #21 and HODrk #22). E) wild type (FSO35-2A and FSO35-4A) *RHO5*^*G12V*^ (FSO88-3A and FSO88-2B) *ras2* (FSO98-6B and FSO98-3A) *RHO5*^*G12V*^ *ras2* (FSO78-14B and FSO78-6C).

Next, we addressed the relationship between Rho5 and the cAMP/PKA signaling pathway. Since growth of mutants lacking components of that pathway is generally not impaired under standard growth conditions, we employed their sensitivity towards hydrogen peroxide as a readout for epistasis analyses with *rho5* deletions. Although PKA also phosphorylates several cytosolic enzymes involved in carbohydrate metabolism, its major effect on nuclear gene expression under these conditions is mediated by the redundant transcription factors Msn2/Msn4 (Fig 1; reviewed in [4,50]). As they also mediate the yeasts general stress response, it is not surprising that *msn2 msn4* double deletions display an increased sensitivity towards oxidative stress exerted by hydrogen peroxide (Fig 5A). This phenotype cannot be rescued by an additional *rho5* deletion, which on its own shows hyper-resistance, indicating that Rho5 acts upstream of Msn2/Msn4 in the signaling cascade.

**Table 2:** Yeast strains employed in this work.

Designation	Genotype	Reference
HD56-5A	*MATalpha ura3–52 his3–11,15 leu2–3,112 MAL3 SUC2 GAL*	[90]
HLBO37-4D	*MATalpha ura3–52 his3–11,15 leu2–3,112 RHO5* ^*G12V*^ *-SkHIS3*	[83]
FSO105-1B	*MATalpha ura3–52 leu2–3,112 his3–11,15 bcy1::KlLEU2*	this work
FSO105-1C	*MATalpha ura3–52 leu2–3,112 his3–11,15 rho5::kanMX*	this work
FSO105-1C	*MATalpha ura3–52 leu2–3,112 his3–11,15 rho5::kanMX*	this work
FSO105-1D	*MATa ura3–52 leu2–3,112 his3–11,15 rho5::kanMX bcy1::KlLEU2*	this work
FSO105-4A	*MATa ura3–52 leu2–3,112 his3–11,15 bcy1::KlLEU2*	this work
FSO105-7C	*MATalpha ura3–52 leu2–3,112 his3–11,15 rho5::kanMX bcy1::KlLEU2*	this work
FSO105-7D	*MATa ura3–52 leu2–3,112 his3–11,15 rho5::kanMX*	this work
FSO105-7D	*MATa ura3–52 leu2–3,112 his3–11,15 rho5::kanMX*	this work
FSO105-7D	*MATa ura3–52 leu2–3,112 his3–11,15 rho5::kanMX*	this work
FSO16-3B	*MATa ura3–52 his3–11,15 leu2–3,112*	this work
FSO35-1A	*MATalpha ura3–52 his3–11,15 leu2–3,112 rho5::kanMX*	this work
FSO35-2A	*MATa ura3–52 his3–11,15 leu2–3,112*	this work
FSO35-4A	*MATalpha ura3–52 his3–11,15 leu2–3,112*	this work
FSO36-11A	*MATalpha ura3–52 his3–11,15 leu2–3,112*	this work
FSO36-11B	*MATa ura3–52 his3–11,15 leu2–3,112 msn2::KlLEU2 msn4::KlLEU2*	this work
FSO36-11C	*MATalpha ura3–52 his3–11,15 leu2–3,112 rho5::kanMX msn2::KlLEU2 msn4::KlLEU2*	this work
FSO36-11D	*MATa ura3–52 his3–11,15 leu2–3,112 rho5::kanMX*	this work
FSO36-5A	*MATalpha ura3–52 his3–11,15 leu2–3,112 msn2::KlLEU2 msn4::KlLEU2*	this work
FSO36-5B	*MATa ura3–52 his3–11,15 leu2–3,112*	this work
FSO36-5C	*MATa ura3–52 his3–11,15 leu2–3,112 rho5::kanMX msn2::KlLEU2 msn4::KlLEU2*	this work
FSO36-5D	*MATalpha ura3–52 his3–11,15 leu2–3,112 rho5::kanMX*	this work
FSO43-1D	*MATa ura3–52 his3–11,15 leu2–3,112 rho5::SpHIS5*	this work
FSO55-9A	*MATa ura3–52 his3–11,15 leu2–3,112*	this work
FSO55-9B	*MATalpha ura3–52 his3–11,15 leu2–3,112*	this work
FSO56-1A	*MATa ura3–52 his3–11,15 leu2–3,112 rho5::SpHIS5*	this work
FSO56-1B	*MATalpha ura3–52 leu2–3,112 his3–11,15 hxk1::KlLEU2*	this work
FSO56-1C	*MATa ura3–52 leu2–3,112 his3–11,15 hxk2::kanMX*	this work
FSO56-1D	*MATalpha ura3–52 his3–11,15 leu2–3,112 rho5::SpHIS5 hxk1::KlLEU2 hxk2::kanMX*	this work
FSO56-2A	*MATalpha ura3–52 his3–11,15 leu2–3,112 rho5::SpHIS5*	this work
FSO56-2B	*MATa ura3–52 his3–11,15 leu2–3,112 rho5::SpHIS5 hxk1::KlLEU2*	this work
FSO56-3A	*MATa ura3–52 leu2–3,112 his3–11,15 hxk1::KlLEU2*	this work
FSO56-3C	*MATalpha ura3–52 leu2–3,112 his3–11,15 hxk2::kanMX*	this work
FSO56-4A	*MATalpha ura3–52 his3–11,15 leu2–3,112 rho5::SpHIS5 hxk1::KlLEU2*	this work
FSO56-4B	*MATa ura3–52 his3–11,15 leu2–3,112 rho5::SpHIS5 hxk2::kanMX*	this work
FSO56-4D	*MATalpha ura3–52 leu2–3,112 his3–11,15 hxk1::KlLEU2 hxk2::kanMX*	this work
FSO56-6D	*MATa ura3–52 his3–11,15 leu2–3,112 rho5::SpHIS5 hxk1::KlLEU2 hxk2::kanMX*	this work
FSO56-8D	*MATa ura3–52 leu2–3,112 his3–11,15 hxk1::KlLEU2 hxk2::kanMX*	this work
FSO56-9C	*MATalpha ura3–52 his3–11,15 leu2–3,112 rho5::SpHIS5 hxk2::kanMX*	this work
FSO62-7A	*MATalpha ura3–52 his3–11,15 leu2–3,112 rho5::SpHIS5*	this work
FSO66-3C	*MATa ura3–52 his3–11,15 leu2–3,112 reg1::kanMX*	this work
FSO67-2C	*MATalpha ura3–52 leu2–3,112 his3–11,15 rho5::KlURA3 snf1::SpHIS5*	this work
FSO71-10B	*MATa ura3–52 his3–11,15 leu2–3,112 reg1::kanMX*	this work
FSO71-15A	*MATalpha ura3–52 leu2–3,112 his3–11,15 rho5::KlURA3*	this work
FSO71-15B	*MATa ura3–52 leu2–3,112 his3–11,15 reg1::kanMX snf1::SpHIS5*	this work
FSO71-15D	*MATalpha ura3–52 leu2–3,112 his3–11,15 rho5::KlURA3 reg1::kanMX*	this work
FSO71-1A	*MATa ura3–52 his3–11,15 leu2–3,112*	this work
FSO71-1B	*MATa ura3–52 leu2–3,112 his3–11,15 rho5::KlURA3 snf1::SpHIS5*	this work
FSO71-1D	*MATalpha ura3–52 his3–11,15 leu2–3,112 reg1::kanMX*	this work
FSO71-2A	*MATa ura3–52 leu2–3,112 his3–11,15 rho5::KlURA3*	this work
FSO71-2C	*MATalpha ura3–52 leu2–3,112 his3–11,15 reg1::kanMX snf1::SpHIS5*	this work
FSO71-5D	*MATa ura3–52 his3–11,15 leu2–3,112 snf1::SpHIS5*	this work
FSO71-6B	*MATalpha ura3–52 leu2–3,112 his3–11,15 rho5::KlURA3 snf1::SpHIS5*	this work
FSO71-7B	*MATalpha ura3–52 his3–11,15 leu2–3,112*	this work
FSO71-7C	*MATa ura3–52 leu2–3,112 his3–11,15 rho5::KlURA3 reg1::kanMX snf1::SpHIS5*	this work
FSO71-9B	*MATa ura3–52 leu2–3,112 his3–11,15 rho5::KlURA3 reg1::kanMX snf1::SpHIS5*	this work
FSO71-9C	*MATalpha ura3–52 his3–11,15 leu2–3,112 snf1::SpHIS5*	this work
FSO71-9D	*MATalpha ura3–52 leu2–3,112 his3–11,15 rho5::KlURA3 reg1::kanMX*	this work
FSO75-4D	*MATa ura3–52 leu2–3,112 his3–11,15 rho5::kanMX*	this work
FSO75-7D	*MATalpha ura3–52 leu2–3,112 his3–11,15 rho5::kanMX*	this work
FSO78-14B	*MATa ura3–52 leu2–3,112 his3–11,15 RHO5* ^*G12V*^ *::SkHIS3 ras2::SkHIS3*	this work
FSO78-6C	*MATalpha ura3–52 leu2–3,112 his3–11,15 RHO5* ^*G12V*^ *::SkHIS3 ras2::SkHIS3*	this work
FSO79-4C	*MATa ura3–52 leu2–3,112 his3–11,15 reg1::kanMX mig1::SkHIS3*	this work
FSO79-7C	*MATa ura3–52 leu2–3,112 his3–11,15 reg1::kanMX*	this work
FSO79-8C	*MATalpha ura3–52 leu2–3,112 his3–11,15 reg1::kanMX mig1::SkHIS3*	this work
FSO84-10B	*MATalpha ura3–52 his3–11,15 leu2–3,112 rim15::KlLEU2 mck1::KlLEU2*	this work
FSO84-10D	*MATa ura3–52 his3–11,15 leu2–3,112 rho5::SpHIS5 rim15::KlLEU2 mck1::KlLEU2*	this work
FSO84-3D	*MATalpha ura3–52 his3–11,15 leu2–3,112 rho5::SpHIS5 rim15::KlLEU2 mck1::KlLEU2*	this work
FSO84-4A	*MATa ura3–52 his3–11,15 leu2–3,112 rim15::KlLEU2 mck1::KlLEU2*	this work
FSO86-1C	*MATalpha ura3–52 his3–11,15 leu2–3,112 rim15::KlLEU2*	this work
FSO86-2A	*MATalpha ura3–52 his3–11,15 leu2–3,112*	this work
FSO86-2C	*MATalpha ura3–52 his3–11,15 leu2–3,112 rho5::kanMX rim15::KlLEU2*	this work
FSO86-6B	*MATa ura3–52 his3–11,15 leu2–3,112 rim15::KlLEU2*	this work
FSO86-7A	*MATa ura3–52 his3–11,15 leu2–3,112*	this work
FSO86-7C	*MATa ura3–52 his3–11,15 leu2–3,112 rho5::kanMX rim15::KlLEU2*	this work
FSO88-1C	*MATalpha ura3–52 leu2–3,112 his3–11,15 RHO5* ^*G12V*^ *::SkHIS3 yak1::KlLEU2*	this work
FSO88-2A	*MATa ura3–52 leu2–3,112 his3–11,15 RHO5* ^*G12V*^ *::SkHIS3 yak1::KlLEU2*	this work
FSO88-2B	*MATalpha ura3–52 leu2–3,112 his3–11,15 RHO5* ^*G12V*^ *::SkHIS3*	this work
FSO88-2C	*MATa ura3–52 leu2–3,112 his3–11,15 yak1::KlLEU2*	this work
FSO88-3A	*MATa ura3–52 leu2–3,112 his3–11,15 RHO5* ^*G12V*^ *::SkHIS3*	this work
FSO88-6A	*MATalpha ura3–52 leu2–3,112 his3–11,15 yak1::KlLEU2*	this work
FSO90-1A	*MATa ura3–52 his3–11,15 leu2–3,112 rho5::KlURA3 mig1::SkHIS3*	this work
FSO90-2A	*MATalpha ura3–52 his3–11,15 leu2–3,112 mig1::SkHIS3*	this work
FSO90-3D	*MATalpha ura3–52 his3–11,15 leu2–3,112 rho5::KlURA3 reg1::kanMX mig1::SkHIS3*	this work
FSO90-6D	*MATalpha ura3–52 his3–11,15 leu2–3,112 rho5::KlURA3 reg1::kanMX mig1::SkHIS3*	this work
FSO90-7A	*MATa ura3–52 his3–11,15 leu2–3,112 mig1::SkHIS3*	this work
FSO90-8B	*MATalpha ura3–52 his3–11,15 leu2–3,112 rho5::KlURA3 mig1::SkHIS3*	this work
FSO98-3A	*MATalpha ura3–52 his3–11,15 leu2–3,112 ras2::SkHIS3*	this work
FSO98-6B	*MATa ura3–52 his3–11,15 leu2–3,112 ras2::SkHIS3*	this work
HOD257-2B	*MATalpha ura3–52 leu2–3,112 his3–11,15 hxk1::KlLEU2*	this work
HOD320-2D	*MATa ura3–52 his3–11,15 leu2–3,112 rho5::kanMX ras2::SkHIS3*	this work
HOD320-6A	*MATalpha ura3–52 his3–11,15 leu2–3,112 ras2::SkHIS3*	this work
HOD343-2A	*MATalpha ura3–52 his3–11,15 leu2–3,112 mck1::KlLEU2*	this work
HOD343-2C	*MATa ura3–52 his3–11,15 leu2–3,112 rho5::SpHIS5 mck1::KlLEU2*	this work
HOD343-4A	*MATalpha ura3–52 his3–11,15 leu2–3,112 mck1::KlLEU2*	this work
HOD343-4C	*MATalpha ura3–52 his3–11,15 leu2–3,112 rho5::SpHIS5 mck1::KlLEU2*	this work
HOD610-2C/ RAS2^G19V^	*MATalpha ura3–52 his3–11,15 leu2–3,112 RAS2* ^*G19V*^ *-KlURA3*	this work
HOD610-1D/ RAS2^G19V^	*MATa ura3–52 his3–11,15 leu2–3,112 RAS2* ^*G19V*^ *-KlURA3*	this work
HODrk21	*MATa ura3–52 his3–11,15 leu2–3,112 RAS2* ^*G19V*^ *-SkHIS3 rho5::kanMX*	this work
HODrk22	*MATa ura3–52 his3–11,15 leu2–3,112 RAS2* ^*G19V*^ *-SkHIS3 rho5::kanMX*	this work
HOD675/ IME1	*MATa/MATalpha ura3–52/ura3–52 his3–11,15/his3–11,15 leu2–3,112/leu2–3,11 RHO5/rho5::KlURA3 REG1/reg1::kanMX RAS2/RAS2*^*G19V*^*-SkHIS3* pJJH3540 (*2 µm-LEU2-PFK2p-IME1*)	this work
HOD677/ IME1	*MATa/MATalpha ura3–52/ura3–52 his3–11,15/his3–11,15 leu2–3,112/leu2–3,11 RHO5/rho5::KlURA3 REG1/reg1::kanMX RAS2/RAS2*^*G19V*^*-SkHIS3* pJJH3540 (*2 µm-LEU2-PFK2p-IME1*)	this work

All strains were derived from HD56-5A [90] and are isogenic except for the mating type and the genetic manipulations indicated.

To further identify at which stage Rho5 interferes with the signaling cascade, we consecutively deleted genes encoding other upstream components. Besides being a direct target for inhibition by PKA-mediated phosphorylation, Msn2/Msn4 can be activated by the protein kinases Rim15, Mck1, and Yak1, which are inhibited by PKA-mediated phosphorylation [51]. Growth curves of the respective mutants revealed that strains lacking Yak1 grew similar to wild-type in the presence and absence of hydrogen peroxide, indicating that this kinase does not mediate Rho5-dependent growth effects (S6 Fig). Yet, a *rim15* deletion caused a moderate increase in sensitivity towards hydrogen peroxide as compared to wild-type cells, whereas *mck1* deletions were clearly hyper-sensitive, with an additive effect in the *rim15 mck1* double deletions (Fig 5B). An additional lack of Rho5, which by itself causes a distinct hyper-resistance towards the stressor, did not lead to a pronounced improvement of growth of the triple *rim15 mck1 rho5* mutants under oxidative stress. This suggests that Rho5 also acts upstream of, but clearly through, the two kinases.

In the presence of glucose and under standard growth conditions the protein kinase A subunits (Tpk1, Tpk2, Tpk3), placed further upstream in the cAMP/PKA signaling cascade, are kept inactive by association with the regulatory subunits encoded by *BCY1* in a heterotetrameric conformation. We thus constructed *bcy1* deletions to obtain strains with a constitutively active PKA. These displayed a pronounced hyper-sensitivity towards hydrogen peroxide, which could not be rescued by an additional *rho5* deletion (Fig 5C).

A major component activating the yeast adenylate cyclase and thereby PKA is another small GTPase, Ras2. To confirm the results obtained with the *bcy1* deletion, we therefore expressed a hyper-active *RAS2*^*G19V*^ allele, which caused an increased sensitivity towards hydrogen peroxide compared to the wild type, again not influenced by the presence or absence of Rho5 (Fig 5D). *Vice versa*, a *ras2* deletion clearly led to an increase in oxidative stress resistance. The latter was not suppressed by expression of a hyper-active *RHO5*^*G12V*^ allele, which leads to a hyper-sensitive phenotype by itself (Fig 5E). Taken together, these data demonstrate that Rho5 acts upstream of the cAMP-dependent PKA and Ras2 in the yeasts nutrient and general stress response.

**Fig 6:**
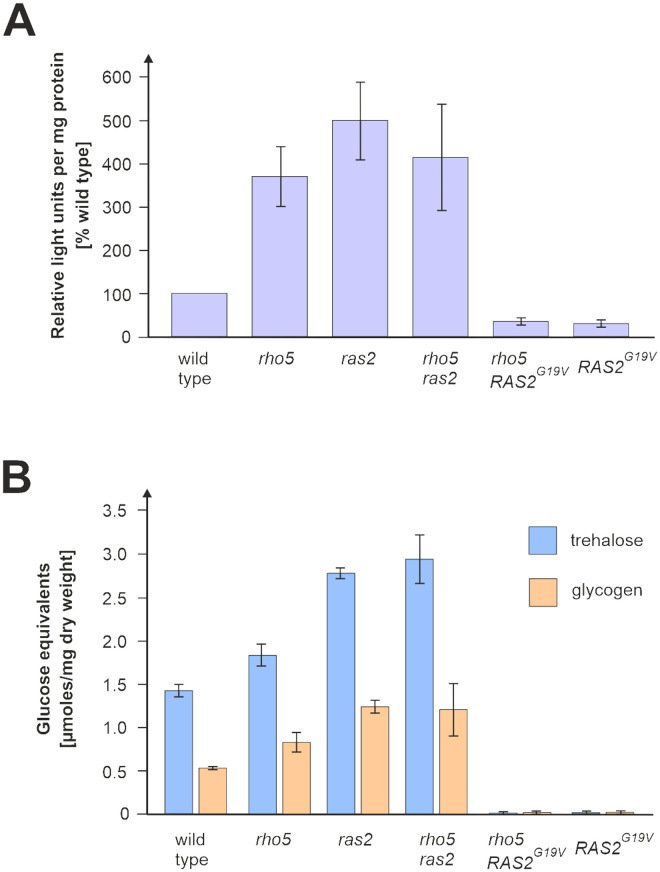
PKA activity and content of reserve carbohydrates in different mutant combinations of *RHO5* and *RAS2.* A) The competition of PKA with luciferase for the ATP substrate was employed to indirectly determine the relative specific activity of PKA in crude extracts of strains with the indicated genotypes. Relative light units produced by the luciferase per mg protein were determined from two biological and two technical replicates, each, setting the wild-type to 100%. Note the inverse relationship to PKA activity: the higher the relative light units generated per mg protein, the lower is the PKA activity. B) Trehalose and glycogen contents were determined in cell lysates from the same strains as above in the early stationary phase, after growth for 22 h in synthetic medium with 2% glucose, and expressed as glucose equivalents liberated upon treatment with trehalase or amylase, respectively. Two biological and two technical replicates were measured. Strains used were: wild type (HD56-5A), *rho5* (FSO35-1A), *ras2* (HOD320-6A), *rho5 ras2* (HOD320-2D), *rho5 RAS2*^*G19V*^ (HOD610-2C/RAS2^G19V^), *RAS2*^*G19V*^ (HODrk22).

In line with these findings and according to the working model proposed in Fig 1, Rho5 would act as a positive regulator of Ras/cAMP signaling. As a consequence, one would expect strains with a *rho5* deletion to have a decreased PKA activity, causing an accumulation of glycogen and trehalose in these cells as compared to wild-type. As shown in Fig 6, these predictions were experimentally confirmed. Thus, PKA activity in crude extracts, as reflected by the inverse relationship to relative light units produced in a luciferase-based assay, was indeed decreased by approximately 4-fold in the *rho5* deletion as compared to the wild-type (Fig 6A). A similar decrease was observed in the *ras2* deletion employed as a control and could not be relieved in the *ras2 rho5* double deletion. In contrast, the hyper-active *RAS2*^*G19V*^ allele led to an elevated PKA activity which was not altered by a *rho5* deletion Fig 6A. Accordingly, the same set of strains showed a strong accumulation of both glycogen and trehalose in strains lacking either Ras2 or Rho5, or both, whereas in strains with the hyper-active *RAS2*^*G19V*^ allele these carbohydrates ranged at the limit of detection Fig 6B. Together, these findings support a positive regulatory role of Rho5 upstream of Ras2 in cAMP-mediated glucose signaling.

## Discussion

Following previous hints for a participation of the small yeast GTPase Rho5 in the response to nutrient availability [35] a more detailed analysis of its crosstalk with the known glucose signaling pathways was initiated in this work. In order to substantiate the physiological relevance of the Rho5 molecular switch, we started by global analyses of both the transcriptome and the proteome in a *rho5* deletion *versus* a wild-type strain, which will first be discussed.

### Global expression analyses

Given that Rho5 was first described as a negative regulator of cell wall integrity (CWI) signaling [36], it was not surprising to find the upregulation of genes and/or proteins that influence the architecture of the cell surface in a *rho5* deletion. This extends to eisosomal proteins, as their turnover was shown to be mediated by Slt2, the downstream mitogen-activated protein kinase (MAPK) of the CWI pathway [52]. Likewise, an increase in the concentration of mitochondrial proteins and/or the expression of their encoding genes was detected. This can be explained by the participation of wild-type Rho5 in stress-dependent mitophagy, resulting in a reduced rate of mitophagy in the deletion mutant, which should affect retrograde signaling for nuclear gene expression [53,54].

Of major interest in the context of this work was the identification of a number of genes and proteins involved in carbohydrate metabolism that were upregulated in the absence of Rho5. Thus, various hexose transporters, encoded by *HXT* genes, increased in gene expression, protein concentration, or both. These comprised the high-affinity transporters Hxt6/Hxt7, whose almost identical sequences prohibit their differential analysis by mass spectrometry [55], and Hxt5 with a moderately high affinity for glucose. All three genes are expressed at low glucose concentrations, as confirmed by transcriptome analyses in wine fermentations [3,56]. The concentration of other Hxt transporters (Hxt13, 15, 16, 17; again not clearly differentiated in proteome analyses due to their high sequence similarities), but not the expression of their encoding genes, was also elevated in a *rho5* deletion. As these proteins are primarily associated with transport of polyols rather than hexose import [57], we speculate that their turnover could be decreased under osmotic or oxidative stress. In contrast, the expression of the *HXT11* gene, encoding another member of the transporter family which is highly similar to Hxt9, was found to be significantly downregulated in a *rho5* deletion. This could be due to the role of this transporter in pleiotropic drug resistance, and to a different set of transcription factors, Pdr1 and Pdr3, which control its gene expression [58]. Finally, the gene encoding the hexokinase isoform 1, *HXK1*, as well as the one for glucokinase, *GLK1*, both subject to repression at high glucose concentrations as discussed for the *HXT* genes [48], were also found to be upregulated both at the transcriptional and the protein level in a *rho5* deletion. This suggested a general role of Rho5 in glucose repression which was supported by our data on epistatic relationships and respiratory capacity, as discussed below.

Regarding the pentose phosphate pathway (PPP), it is believed to contribute only to a minor extent to glucose metabolism in *S. cerevisiae* as compared to other yeasts (reviewed in [59]). Nevertheless, its oxidative part, especially the reaction of glucose-6-phosphate dehydrogenase (Zwf1), produces reduction power (NADPH) required to cope with oxidative stress [60–62]. Rho5 also contributes to a proper response to such stress [39,40,63]. Interestingly, a lack of Rho5 leads to an increased expression of the genes encoding some of the apparently less relevant isozymes acting in the pathway, like *SOL4*, *GND2*, and *TKL2* [64–66]. *NQM1*, which encodes a putative transaldolase isozyme with a low catalytic activity [67], is repressed by glucose, and was proposed to regulate transcription of genes involved in stress resistance in stationary phase cells [68]. One could thus assume that while the major isozymes in the PPP catalyze the housekeeping reactions, alternative isozymes are upregulated to cope with stressful conditions such as glucose depletion and the presence of hydrogen peroxide, a response which may involve the Rho5 molecular switch.

Finally, we found that genes and enzymes related to the biosynthesis of the stress-protectant disaccharide trehalose and the turnover of the reserve polysaccharide glycogen, both in its synthesis and its degradation, are upregulated in a *rho5* deletion as compared to a wild-type strain (see [69] for a comprehensive review on yeast reserve carbohydrate metabolism). Both compounds accumulate while yeast cells are approaching stationary phase from a glucose-rich medium [70], and were found to be significantly reduced in cells lacking Rho5 in the stationary phase [51]. In contrast, we here clearly show that both trehalose and glycogen accumulate in a *rho5* deletion in our strain background, with a concomitant decrease in PKA activity. The apparent contradiction in the accumulation of reserve carbohydrates can be explained by the different time points at which these parameters have been determined. We here left the cells to grow for only 22 h in fresh medium, i.e., just reaching stationary phase and not expected to commence with the degradation of trehalose and glycogen [70], whereas in the previous work cells were only harvested after 72 h [51]. Nevertheless, these findings underline the important role of Rho5 in regulating the cells response to both nutrient limitation and oxidative stress [35] which are reflected in our transcriptome and proteome analyses.

### Interactions of Rho5 with Hxk2- and SNF1-dependent glucose signaling

The apparent upregulation of many hexose transporters and genes subject to glucose repression observed in the transcriptome and proteome analyses prompted a deeper analysis of the genetic interactions of *RHO5* with genes encoding components in the known glucose signaling pathways depicted in Fig 1. As expected from previous reports, *hxk2* null mutants displayed only a mild growth defect, attributed to the fact that yeast has two further kinases capable of glucose phosphorylation, Hxk1 and Glk1 [49]. Although the latter are usually not expressed in glucose media due to catabolite repression, this regulation is alleviated by a lack of Hxk2, thus restoring the ability to channel glucose into glycolysis [45,48]. Glucose signaling is exerted by Hxk2 in two ways: i) It inhibits activation of the SNF1 complex, depending on the rate of glucose phosphorylation. ii) It can enter the nucleus and act as a coactivator of the Mig1 repressor (reviewed in [47]). The improvement in growth of a *hxk1 hxk2* double deletion by an additional lack of Rho5 could be attributed to the observed effect of the GTPase on respiration, which itself is subject to glucose repression in *S. cerevisiae* [71,72]. Apparently, wild-type Rho5 contributes to this repression, as it is partially alleviated in the *rho5* deletion. The limited amount of glucose phosphorylated by the remaining glucokinase in the triple deletion strains, originally observed in a *RHO5* wild-type background, could then be more efficiently used for ATP generation by an increased rate of respiration in the *rho5* null mutant compared to the wild-type, as found herein. Thus, *rho5* deletions display a lower rate of mitophagy [39,40], which may result in the presence of more (although partially defective) mitochondria. The latter would continue to respire, leading to an enhanced ATP supply which then could counteract some of the slow growth defects of the hexokinase double mutants. Our data also indicate that too little Rho5 more strongly affects respiratory capacity than too much, as the hyper-active *RHO5*^*G12V*^ allele did not show the expected decrease.

With regard to signaling through the SNF1 complex we found that a lack of the kinase activity causes a growth defect independent of Rho5. On the other hand, hyper-activation of the complex, achieved by deletion of the gene encoding the inhibitory Reg1 phosphatase, also severely impaired growth, but was aggravated by a lack of Rho5. Further epistasis analyses then showed that the slow growth of the *reg1* deletion depended on the upregulation of the common SNF1 pathway, which is frequently mediated by the transcriptional repressor Mig1 (Fig 1; [11]). However, the genetic interaction between *RHO5* and *REG1* required only SNF1 activity, but not Mig1. In this context, an indirect effect of Rho5 on growth by its signaling to cell wall synthesis through SNF1 can be excluded, as this would also be mediated by Mig1 [17,18]. The observed similarity in reduced growth of *reg1 rho5* double deletions as in the *reg1 ras2* mutants indicate that it is either the cross-regulation of SNF1 and PKA or the regulation of the Msn2/Msn4 transcription factor through both routes that accounts for the growth phenotypes. This scenario is further substantiated by the observation that the slow growth of *rho5 reg1* double deletions can be suppressed by a hyper-active Ras2^G19V^.

### Interactions of Rho5 with cAMP/PKA signaling

To facilitate the epistasis analyses between *rho5* and mutants defective in cAMP/PKA signaling we employed their effect on oxidative stress response: While strains lacking Rho5 are more resistant towards hydrogen peroxide than wild type, blocks in the cAMP/PKA pathway cause an increased sensitivity [39,40,73]. The latter would be expected to be rescued in double mutants with *rho5* if the GTPase acted in a downstream step of the signaling pathway or in a parallel route. On the other hand, cells would be expected to remain hyper-sensitive if Rho5 was acting upstream of the block in the same signaling pathway. Moreover, opposite results would be expected when hyper-active mutant alleles were employed in the epistasis analyses. For example, the hyper-sensitivity of a Rho5^G12V^ variant towards hydrogen peroxide would be rescued by blocking a step downstream in the same, but not a different, signaling pathway. Under these premisses, our results clearly show that *RHO5* is epistatic to *RAS2* and all genes encoding downstream components of the cAMP/PKA pathway depicted in Fig 1. Interestingly, the signal to PKA elicited by Rho5 under oxidative stress seems to be transmitted to the transcription factors Msn2/Msn4 predominantly by the branch inactivating the Rim15/Mck1 kinases, as the hyper-sensitivity of the double deletion mutants cannot be rescued by a *rho5* deletion. Note that the degree of resistance mediated by the two kinases is additive, with our data suggesting that Mck1 plays a more prominent role in this case. These findings are in line with previous reports indicating that the two kinases mediate Rho5-dependent survival under glucose starvation [51]. As survival upon starvation was suggested to be compromised by a decreased activity of Ras2, causing the production of reactive oxygen species (ROS) [74], this could be explained by our finding that Rho5 acts upstream of Ras2 in glucose signaling. Similar to our results, Rim15, but not a third protein kinase Yak1, was also found to differentially control gene expression of *TPK1*, one of the three PKA catalytic subunits, under heat shock and salt stress [75]. Moreover, we previously reported that a *rho5* deletion displays severe synthetic growth defects not only with *gpr1* and *gpa2* deletions affecting the other branch of glucose signaling to PKA (Fig 1), but also with a *sch9* deletion [35]. The latter lacks a protein kinase required to mediate nutrient signaling by TORC1, which inhibits Rim15 by phosphorylation, and thus integrates general nutrient with glucose signaling to fine-tune the activity of Msn2/Msn4 and thereby the expression of their target genes [76,77]. A feedback regulatory loop is then provided by Tps2, a trehalose-6-phosphate phosphatase, whose gene expression is under the control of Msn2/Msn4. *TPS2* expression was found to be upregulated in our RNAseq data and confirmed by the mass-spectrometry data in a *rho5* deletion. The phosphatase then activates Rim15 by removal of inhibitory phosphate groups [78].

### To bind it all – a hypothesis for the role of Rho5 in glucose signaling

The data presented in this work and discussed above strongly indicate that the function of Rho5 in transmission of the glucose availability in the medium into a cellular response is largely mediated by PKA-dependent signaling to the transcription factors Msn2/Msn4. We pinpointed Rho5 to activate a step upstream of Ras2, with further signaling executed via the Rim15/Mck1 branch of protein kinases (Fig 1). This holds true at least for the parallel response to oxidative stress, based on the epistasis analyses with deletion mutants within the cAMP/PKA pathway. As stress-related genes show an increased expression in cells lacking Rho5, the GTPase should activate the pathway in wild-type cells under standard growth conditions in glucose-rich media. How does this fit with the general expression analyses and the results on epistasis analyses for hexokinase and the SNF1 complex depicted in the left-hand part of Fig 1?

The transcription factors Msn2/Msn4 are not only a target of phosphorylation for PKA, but in fact have first been identified as multicopy suppressors of a temperature-sensitive *snf1* mutant, as they are also inactivated by SNF1-dependent phosphorylation [79]. Moreover, an inverse relationship was found for SNF1 and PKA activities, as one inhibits the other (reviewed in [80]). Both, a lack of kinase activity in the *snf1* deletion, as well as a constitutively high activity in the *reg1* deletion reduce the ability to grow on glucose, as reported earlier in other strain backgrounds [81,82]. We found this growth retardation to be aggravated in *reg1 rho5* and in *reg1 ras2* double deletions, which could be explained by a further increase in kinase activity caused by the lack of the feedback inhibition between PKA and SNF1. Consequently, Msn2/Msn4-dependent gene expression would be strongly inhibited in a manner independent of Mig1, as observed. In contrast, the growth retardation observed in single *snf1* mutants and the *snf1 rho5* double deletion strains may be attributed to deregulation of Mig1-mediated repression.

Finally, the regulation through PKA and SNF1 also serves to embed Rho5 action in mitochondrial turnover and thereby in energy metabolism. We recently reported on the genetic interaction between *RHO5* and *POR1*, a gene encoding the major voltage-dependent anion channel (VDAC) in *S. cerevisiae* [83]. In turn, the VDAC is required to regulate the activity and nuclear localization of the SNF1 complex [84,85]. Autophagy, and specifically mitophagy, are also interconnected at multiple levels with these signaling pathways. For instance, PKA inhibits autophagy by phosphorylation of Msn2/Msn4 [34]. This function is coordinated with the response to other nutrient limitations mediated by the TORC1 complex and the protein kinase Sch9 and its activation of Rim15 [34]. Interestingly, a *rho5* deletion is synthetically lethal with *sch9* null mutants, also indicating a concerted action of Rho5 through PKA and TORC1 signaling [35]. And the activated SNF1 complex under glucose limitation also activates TORC1, ultimately promoting expression of Msn2/Msn4-dependent genes [86]. These include *ATG8* and *ATG39*, which encode components of the autophagic machinery [87,88]. Another autophagy component, Atg21, was shown to interact directly with Rho5 [63]. As Rho5 rapidly localizes to mitochondria under oxidative stress together with its dimeric GEF Dck1/Lmo1, it has been proposed to directly trigger mitophagy [83,89]. Whether or not this is related to its effect on Ras2 observed herein remains to be determined, given that Ras2 and PKA in its inactive tetrameric form are also recruited to mitochondria by Hsp60 and regulate their turnover [28].

## Materials and methods

### Yeast strains and growth conditions

Yeast strains used in this work are listed in Table 2 and were derived from HD56-5A, one of the parental strains of the common CEN.PK series [90], or its isogenic diploid DHD5 [91]. For cloning purposes and plasmid amplification, *E. coli* strain DH5α was used (Invitrogen, Karlsruhe, Germany). Standard procedures were followed for genetic manipulations of yeast and plasmid constructions [92]. Complete sequences of all plasmids, modified chromosomal loci, and oligonucleotides employed are available upon request.

Rich medium (YEPD) was based on yeast extract (1% w/v) and peptone (2% w/v), supplemented with 2% glucose (w/v). Synthetic media (SC) contained 0.67% yeast nitrogen base (w/v) supplemented with ammonium sulfate, amino acids and bases as required [92], with 2% glucose (w/v, SCD) as a carbon source. Histidine concentration was raised from 2 to 4 mg/L, if necessary, to record growth curves. *E. coli* cells were grown in LB medium (yeast extract at 0.5% w/v, tryptone at 1% w/v, and sodium chloride at 1% w/v), with the addition of 50 mg/L ampicillin or 25 mg/L kanamycin as required for plasmid selection.

### Genetic manipulations and epistasis analyses

Deletion mutants were obtained by one-step gene replacements, using PCR products obtained with primers generating 40–50 bp of homology flanking the genomic target sequences, with selection for genetic markers as described [93]. For complementation of auxotrophic markers with *KlURA3* (pJJH1286) or *KlLEU2* (pJJH1287) from *Kluyveromyces lactis,* modified plasmids were used, which carried the marker genes flanked by the *TEF2* promoter and the *TEF2* terminator from *Ashbya gossypii* and by two *loxP* sites. Alleles encoding hyper-active variants of the GTPase (*RHO5*^*G12V*^ or *RAS2*^*G19V*^) were inserted at the native genetic loci by substitution of the respective deletion markers, using *SkHIS3* inserted into the respective 3’ non-coding regions as selection marker.

Strains with different deletion or mutant alleles were crossed by standard yeast genetic techniques [92], sporulated on plates with 1% potassium acetate (w/v), and subjected to tetrad analyses on YEPD plates using a Singer MSM400 micromanipulator (Singer Instruments, Somerset, UK). Plates were incubated for 3–4 days at 30°C and scanned for documentation. The images were adjusted for brightness and contrast using ImageJ with the same settings for the entire plate, and colony sizes were determined with the analyze particles function of the program. At least 50 tetrads were separated for each cross and used to compare colony sizes after assigning the genotypes from marker analyses. The averaged sizes of wild-type segregants from each cross were set to 100% and the relative sizes of mutant segregants were averaged and calculated. Statistical analyses were obtained using the T.TEST function of Excel.

In strains carrying the hyper-active *RAS2*^*G19V*^ allele, sporulation does not occur due to the inhibitory action of PKA on the master transcriptional regulator Ime1, which is required for proper meiosis [94]. Therefore, we constructed a yeast 2 µm plasmid based on YEp181 with *LEU2* as a selective marker [95]. The coding sequence of *IME1* was amplified by PCR from a wild-type strain (HD56-5A) with the primer pair 25.028/25.029 (5’-gctcggatcc-ATGCAAGCGGATATGCATGG-3’ and 5’-gcgaaccggtaagcTTAAGAATAGGTTTTACT-AAACTTG-3’; underlined sequences designating the BamHI and HindIII restriction sites used for cloning, letters in small print are not homologous to the target sequence) and cloned under the control of a tailored *PFK2* promotor employed previously [61], to yield plasmid pJJH3540 (complete sequence and a set of similar expression vectors available upon request). Its inheritance in strains HOD675/IME1 and HOD677/IME1 was ensured by selection for leucine prototrophy and the respective diploids were grown overnight in rich medium (YEPD), and sporulated on potassium acetate plates as described above.

Growth curves of yeast strains under standard and oxidative stress conditions were recorded with a Varioscan Lux plate reader (ThermoFisher Scientific) as detailed in [83].

### Determination of reserve carbohydrates and protein kinase A activity

For the determination of glycogen and trehalose content the method of Parrou and Francois [96] was adapted. Thus, cells were grown overnight in 5 ml of YEPD to late logarithmic phase, inoculated to an OD_600_ of 0.3 in 10 ml of of fresh synthetic medium with 2% glucose (SCD) and incubated for another 22 h at 28°C with shaking at 180 rpm in 100 mL Erlenmeyer flasks. The OD_600_ was determined to range between 5–8 and cells from 5 mL of each culture were harvested by centrifugation (3 min at 5000 g at room temperature), drained and suspended in 250 µL of sodium carbonate (250 mM). Suspensions were transferred to screw-capped Eppendorf tubes, tightly closed and incubated for 4 h with shaking (800 rpm) at 95°C. The pH was adjusted by addition of 150 µL of 1 M acetic acid and 600 µL sodium acetate (0.2 M, pH 5.2), yielding a total of 1 mL suspension. Samples were divided into two 500 µL aliquots. For assessment of the glycogen content, 1 U of amyloglucosidase from *Aspergillus niger* (Sigma/Aldrich, A7420) was added and incubated overnight at 57°C with shaking at 800 rpm. The other half of each sample was incubated with 0.05 U of trehalase (Sigma/Aldrich, T8778) at 30°C, also overnight with constant agitation. Prior to determination of the glucose liberated in both assays, tubes were centrifuged for 10 min in a microfuge at full speed and the supernatant was carefully removed into standard 1.5 mL Eppendorf tubes. Depending on the expected glycogen and trehalose content, 10–100 µL were employed for enzymatic determination of glucose. This was done with the glucose determination kit of Roche (Boehringer Mannheim, product number 10716251035), which is based on the reduction of NADP measured at 340 nm with hexokinase and glucose-6-phosphate dehydrogenase as ancillary enzymes, basically following the manufacturers instructions. Different from those instructions, assays were performed by adding the samples to a volume of 700 µL testmix containing all ancillary enzymes and NADP. Absorptions at 340 nm were determined prior to sample addition and again 15 min after incubation at room temperature. Glucose concentrations were calculated from the observed differences in A_340_ with an extinction coefficient for NADPH of 6.223 and normalized assuming that 1 OD_600_ in the original culture equals 0.4 mg/mL of dry weight.

Protein kinase A (PKA) activity was determined in crude extracts using a luciferase-coupled assay. Cells were grown in 2.5 mL YEPD precultures overnight, added to 12.5 mL fresh YEPD medium in an Erlenmeyer flask and incubated with shaking for another 4–5 h at 28°C. Crude extracts were prepared from harvested cells by breaking with glass beads in 50 mM potassium phosphate buffer, pH 7.0 and protein content was determined by Microbiuret, as described in detail in [97]. PKA activities were determined with components of a kit originally designed to measure cAMP concentration (cAMP-Glo Assay, Promega product number V1501). As a modification, only the Kemptid peptide substrate and the luciferase coupling was used from the kit, omitting the addition of cAMP and ancillary PKA. Sample volumes were adjusted to 25 µL in buffer, to which 120 µl of testmix were added in a black 96-well microtiter plate. Light generation was then recorded for 10 min at 30°C using a Varioscan Lux plate reader (ThermoFisher Scientific). Initial velocities in the linear range of the curves were used to calculate relative light emissions per min and normalized to the samples protein content. The value for the wild-type was set to 100% and the relative activity for the other strains was calculated independently for each biological and technical replicate. It is important to note that PKA activity competes with luciferase for the ATP substrate producing an inverse relationship, i.e., the higher the relative light units, the lower is the PKA activity.

For all assays described in this section, two biological and two technical replicates were recorded and mean values and standard deviations were computed.

### High-throughput analyses

RNA preparation, RNA seq and bioinformatic analyses were performed by StarSeq (Mainz, Germany). For this purpose, cells were grown in 50 mL SCD in the absence or presence of 0.8 mM hydrogen peroxide, inoculated from fresh overnight cultures to an OD_600_ of 0.2 and grown for another two generations to an OD_600_ of 0.8 at 28°C with shaking at 180 rpm. Cells were collected by centrifugation, frozen in liquid nitrogen and shipped on dry ice.

Proteomes were obtained from mass spectrometry analyses. Therefore, yeast cells were grown in 25 mL SCD, which were inoculated from a fresh overnight culture to an OD_600_ of 0.2 and allowed to grow for another two generations at 28°C and shaking at 180 rpm. Samples were extracted using the iST kit according to the instructions of the manufacturer (Preomics GmbH, Martinsried, Germany). Mass spectrometry using label-free quantification (LFQ) was performed at the Mass Spectrometry Equipment Center of the Department of Biology/Chemistry at the “CellNanOS” research center of the University of Osnabrück. For this purpose, dried peptides were resuspended in 10 µL LC-Load buffer and 2 µL were used to perform reversed-phase chromatography on a Thermo Ultimate 3000 RSLCnano system connected to a TimsTOF HT mass spectrometer (Bruker Corporation, Bremen) through a Captive Spray Ion source. Peptides were separated on a Aurora Gen3 C18 column (25 cm x 75 µm x 1.6 µm) with CSI emitter (Ionoptics, Australia) at temperature of 40°C. Peptides from the column were eluted via a linear gradient of acetonitrile from 10-35% in 0.1% formic acid (v/v) for 44 min at a constant flow rate of 300 nL/min following a 7 min increase to 50%, and finally, 4 min to reach 85% buffer B. Eluted peptides were then directly electro sprayed into the mass spectrometer at an electrospray voltage of 1.5 kV and 3 L/min dry gas.

The MS settings of the TimsTOF were adjusted to positive Ion polarity with a MS range from 100 to 1700 m/z. The scan mode was set to PASEF. The ion mobility was ramped from 0.7 Vs/cm^2^ to 1.5 in 100 ms. The accumulation time was also set to 100 ms. 10 PASEF ramps per cycle resulted in a duty cycle time of 1.17 s. The target intensity was adjusted to 14,000, the intensity threshold to 1,200. The dynamic exclusion time was set to 0.4 min to avoid repeated scanning of the precursor ions, their charge state was limited from 0 to 5. The resulting data were analyzed with PeaksOnline (BSI, Canada) version 11, employing the corresponding Yeast FASTA databases. Precursors were ranging from 600 to 6,000 Da. As modifications carbamidomethylation (C) and oxidation (M) were chosen. DDA-MBR were performed with MS tolerance of 10 ppm and IM tolerance of 0.05 (1/k0).

The obtained data from RNA-sequencing and mass spectrometry were processed using the standard Excel program. Cut-offs were applied at p-values less than 0.05 and at least a twofold change in expression. Proteins detected by mass spectrometry were only considered, if at least one unique peptide was repeatedly found. It should be noted that while some proteins could not be distinguished in the mass spectrometry data due to their high amino acid similarities (e.g., some members of the hexose transporter family), their encoding RNAs were clearly distinguished by RNAseq, based primarily on differences in the 5’- and 3’-noncoding regions.

### Determination of respiratory capacity

A Seahorse analyzer (Agilent Technologies Deutschland GmbH, Waldbronn, Germany) was employed to determine the respiratory capacity of the different yeast strains with the “Seahorse XF Cell Mito Stress Test” with a modification of the test employed for mammalian cells [98]. It allows the measurement of the oxygen consumption rate in live cells. For the assay of yeast cells, cultures were grown overnight in SCD at 28°C with shaking (180 rpm). After dilution to an OD_600_ of 0.4 they were again incubated to reach an OD_600_ of 0.8. Three biological replicates and at least two technical replicates were determined, with the exception of a *rho5* deletion, where only one technical replicate was obtained for one of the three biological replicates.

### Real-time RT-PCR

Real-time RT-PCR (RT-qPCR) was employed to confirm the data of RNAseq analyses for a subset of representative genes. For this purpose, 5 ml of two overnight cultures of each strain where used to inoculate 50 ml of YEPD and grown to an optical density at 600 nm of 0.8 at 28°C. RNA was isolated according to [99] and purified using the “DNA-free” kit (Thermo Fischer Scientific, Schwerte, Germany). The RNA was transcribed into cDNA using the “iScript Reverse Transcription Supermix for RT-qPCR” (Bio-Rad Laboratories GmbH, Feldkirchen, Germany) by reverse transcriptase polymerase chain reaction (RT-PCR). To identify or exclude possible contaminations of the cDNA, a negative control was performed for each measurement. Therefore, the “iScript No-RT Control Supermix” (Bio-Rad Laboratories GmbH, Feldkirchen, Germany) was used in the cDNA synthesis, which does not contain reverse transcriptase. HPLC-purified primers designed with the software “Primer3” were used for qPCR studies ( [100]; S1 Fig). The qPCR was performed using the “iTaq Universal SYBR Green Supermix” (Bio-Rad Laboratories GmbH, Feldkirchen, Germany) in a qTower 2.0 by Analytik Jena (Jena, Germany) with two technical replicates of each of the two biological replicates. All kits and reaction mixtures were used or prepared according to the manufacturer’s instructions. For each qPCR, the threshold cycle (Ct value) was calculated by the machines integrated software. Data were normalized to actin as a housekeeping control using the 2^-ΔΔC^_T_ method according to [101] and analyzed with freely accessible statistical software (R Core Team 2021 at https://www.R-project.org/ and RStudio Team 2015 at: http://www.rstudio.com/).

## Supporting information

S1 Fig Quantitative eal-time RT-PCR analysis of the expression of some selected genes. Relative Fold-changes (rFC) of expression compared to the wild-type control was calculated for the indicated genes based on 2-^ΔΔCt^ analysis from the Ct values of RT q-PCR using the actin gene (*ACT1*) as a housekeeping reference. Means and standard errors of the mean (error bars) were calculated from two technical and two biological replicates. Primers used for RT q-PCR are listed below. Strains used were HD56-5A as a wild-type and FSO62-7A for the *rho5* deletion.
(PDF)

S2 Fig Epistasis analyses based on the growth of strains carrying mutations in genes encoding components of the SNF1 signaling pathway in combination with *RHO5* variants. Growth curves were recorded on synthetic medium with 2% glucose (SCD; supplemented with 4 mg/L histidine for strains with a histidine auxotrophy) as indicated. Error bars give the standard deviations at each time point obtained from at least two biological and two technical replicates from parallel measurements for each curve (i.e., two independent isogenic segregants were measured, with two independent inoculates, each). Genotypes of the otherwise isogenic strains are listed in Table 2 (main text). Strains employed were **A)** wild type (FSO71-1A and FSO71-7B) *rho5* (FSO71-2A and FSO71-15A) *reg1* (FSO71-10B and FSO71-1D) *snf1* (FSO71-5D and FSO71-9C) *rho5 reg1* (FSO71-9D and FSO71-15D) *rho5 snf1* (FSO71-1B and FSO71-6B) *reg1 snf1* (FSO71-15B and FSO71-2C) *rho5 reg1 snf1* (FSO71-7C and FSO71-9B). **B)** wild type (FSO86-7A and FSO86-2A) *rho5* (FSO75-4D and FSO75-7D) *reg1* (FSO71-10B and FSO71-1D) *mig1* (FSO90-7A and FSO90-2A) *rho5 reg1* (FSO71-9D and FSO71-15D) *rho5 mig1* (FSO90-1A and FSO90-8B) *reg1 mig1* (FSO79-4C and FSO79-8C) *rho5 reg1 mig1* (FSO90-3D and FSO90-6D). **C)** wild type (FSO55-9A and FSO55-9B) *rho5* (FSO56-1A and FSO56-2A) *hxk1* (FSO56-3A and FSO56-1B) *hxk2* (FSO56-1C and FSO56-3C) *rho5 hxk1* (FSO56-2B and FSO56-4A) *rho5 hxk2* (FSO56-4B and FSO56-9C) *hxk1 hxk2* (FSO56-8D and FSO56-4D) *rho5 hxk1 hxk2* (FSO56-6D and FSO56-1D).
(PDF)

S3 Fig Epistasis analyses based on growth of segregants from tetrad analyses on rich medium (YEPD). Four exemplary tetrads are shown, with colored circles designating different combinations of gene deletions as indicated. Colony sizes for each combination (determined from pixel area and given as percentage from wild type set at 100%) were determined from 29 tetrads and quantified in the columns of the diagram at the right (n = total number of segregants obtained for each genotype. Error bars are indicated for each mutant combination. Three asterisks indicate highly significant differences with p-values below 0.001; n.s. = not significant). Diploids analyzed were from the cross of a strain carrying a *reg1 mig1* double deletion (FSO79-8C) with one carrying a *snf1* deletion (HOD201-2D).
(PDF)

S4 Fig Respiration measurement of a wild-type strain (HD56-5A), a *rho5* deletion strain (FSO62-7A) and a *RHO5*^*G12V*^ mutant strain (HLBO37-4D). Significance is indicated by one asterisk, while three asterisks indicate a very high significance. Note that differences between each strain with and without hydrogen peroxide are also highly significant but not highlighted with asterisks here for the sake of clarity.
(PDF)

S5 Fig Epistasis analyses for mutants in *REG1*, *RHO5* and *RAS2* based on growth of segregants from tetrad analyses on rich medium (YEPD). Four exemplary tetrads are shown, each, with colored circles designating different combinations of gene deletions as indicated. Colony sizes for each combination (determined from pixel area and given as percentage from wild type set at 100%) were determined and quantified in the columns of the diagram at the right (n = total number of segregants obtained for each genotype. Error bars are indicated for each mutant combination. Three asterisks indicate highly significant differences with p-values below 0.001; n.s. = not significant). Diploids analyzed were: A) From the cross of a strain carrying a *reg1* deletion (FSO79-7C) with one carrying a *ras2* deletion (HOD320-6A); and B) from the heterozygous diploid strain for *reg1 rho5 RAS2*^*G19V*^ transformed with an *IME1* expression plasmid (HOD666/IME1).
(PDF)

S6 Fig Epistasis analyses of *rho5* and *yak1* mutant combinations based on their sensitivity towards hydrogen peroxide. Growth curves were recorded on synthetic medium with 2% glucose (SCD), as described in material and methods of the main text, with or without hydrogen peroxide as indicated. Error bars give the standard deviations at each time point obtained from at least two biological and two technical replicates. Genotypes of the otherwise isogenic strains are listed in Table 2. Strains employed were wild type (FSO35-2A and FSO35-4A), *RHO5*^*G*^*¹²*^*V*^, (FSO88-3A and FSO88-2B)*, yak1* (FSO88-2C and FSO88-6A), *RHO5*^*G*^*¹²*^*V*^ *yak1* (FSO88-2A and FSO88-1C).
(PDF)

S1 Table Source data on RNA sequencing comparing gene expression in a *rho5* deletion to that of a wild-type grown in synthetic complete medium (SCD). Three biological replicas were recorded with strains and growth conditions explained in the legend of Fig 2 and in the Material and Methods section. For a quick reference, genes have been ordered according to the log2-Fold Change (column D). Gene names are given in column B, statistical significance of changes (p-value) in column G and protein functions according to the Saccharomyces Genome Database (SGD; https://www.yeastgenome.org; last accessed on July 3, 2025) in column L.
(CSV)

S2 Table Source data on RNA sequencing comparing gene expression in a *rho5* deletion to that of a wild-type grown in synthetic complete medium (SCD) in the presence of 0.8 mM hydrogen peroxide. Three biological replicas were recorded with strains and growth conditions explained in the legend of Fig 2 and in the Material and Methods section. For a quick reference, genes have been ordered according to the log2-Fold Change (column D). Gene names are given in column B, statistical significance of changes (p-value) in column G and protein functions according to the Saccharomyces Genome Database (SGD; https://www.yeastgenome.org; last accessed on July 3, 2025) in column L.
(CSV)

S3 Table Source data on mass spectrometry analysis comparing protein amounts in a *rho5* deletion to that of a wild-type grown in synthetic complete medium (SCD). Three biological replicas were recorded with strains and growth conditions explained in the legend of Fig 2 and in the Material and Methods section. For a quick reference, gene names are given in column B, relative changes in protein abundance are given in column C, and protein functions according to the Saccharomyces Genome Database (SGD; https://www.yeastgenome.org; last accessed on July 3, 2025) in column U.
(CSV)
