# Supplementary material for: The small GTPase Rho5—Yet another player in yeast glucose signaling
Source: PLoS Genet. 2025 Sep 9;21(9):e1011858. doi: 10.1371/journal.pgen.1011858 (PMC12440216; doi:10.1371/journal.pgen.1011858)
Supplement: S1 Fig — Relative Fold-changes (rFC) of expression compared to the wild-type control was calculated for the indicated genes based on 2-ΔΔCt analysis from the Ct values of RT q-PCR using the actin gene (ACT1) as a housekeeping reference. Means and standard errors of the mean (error bars) were calculated from two technical and two biological replicates. Primers used for RT q-PCR are listed below. Strains used were HD56-5A as a wild-type and FSO62-7A for the rho5 deletion. (PDF) [file pgen.1011858.s001.pdf]

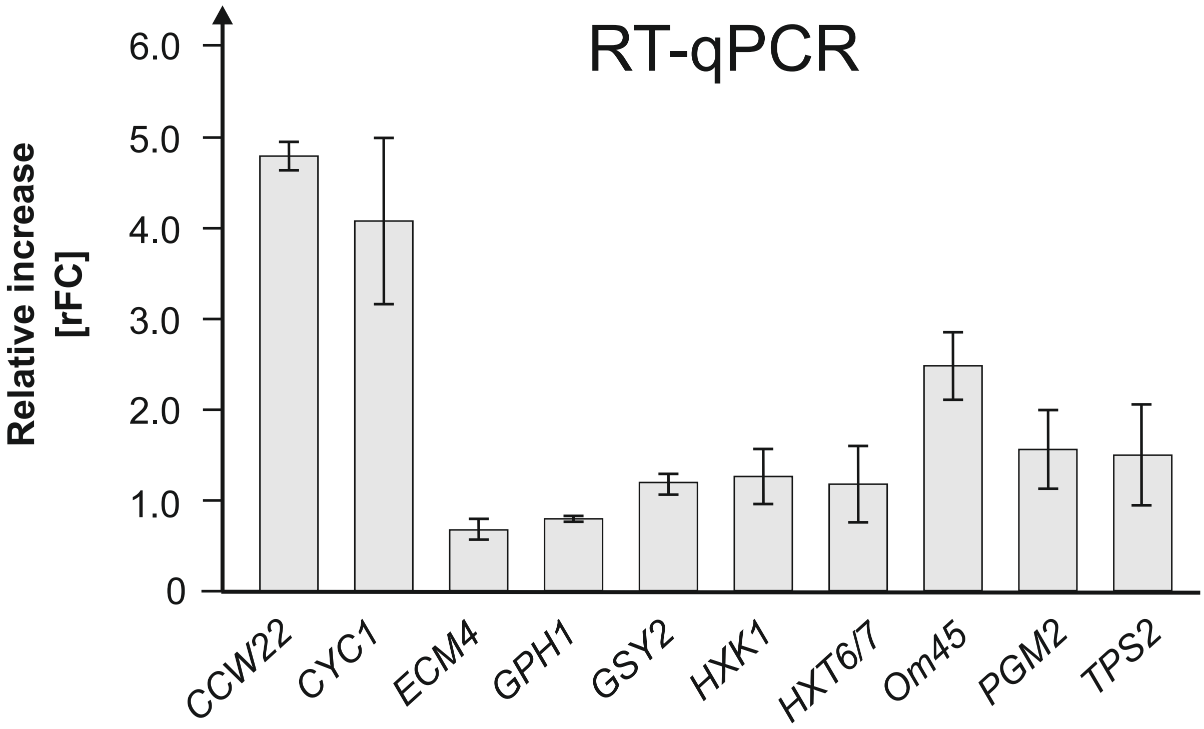

Number	Name	Sequence (5' → 3')
25.030	ECM44_F	CGA GTG GTA CAA ACG GAG CT
25.031	ECM44_R	AGC AGG AAC AAG GTC CGT TT
25.032	CCW22_F	TGC AAT TCT CTA CCG TCG CT
25.033	CCW22_R	TAC AAC AAT AAA GCG GCA GC
25.034	OM45_F	AGAAGC GTT TCG AAG AGG CA
25.035	OM45_R	ATG GCA TCG GAC ACG TTC TT
25.036	CYC1_F	TGA CTG AAT TCA AGG CCG GT
25.037	CYC1_R	ATT AAG TCG TTT CTG TCT TTT TCC T
25.038	GPH1_F	AGC TTG CTT CGT CGA CTC AA
25.039	GPH1_R	AAG GAT GCA GCA CAC CAG AA
25.040	PGM2_F	ACG GCC CAT CTT TCG TTT CT
25.041	PGM2_R	GAG ACT CAT CGG CTG GGA AG
25.042	GSY2_F	GGG GTT ACA CAC CTG CAG AA
25.043	GSY2_R	CAT ATG GGC CAT CGT CGT CA
25.044	TPS2_F	TTG TCA AAG ACC CAG CTG CA
25.045	TPS2_R	TGC CAT GTT GAT GCC AGA CT
25.046	HXT7_F	CGG TCG TAA GGT GGG TTT GA
25.047	HXT7_R	GCA CCC ATG ATC AAA CGC TG
25.048	HXT6_F	CTA TGG GGT GCT GCA TCC AT
25.049	HXT6_R	CGT GAG CCA TTT CTT CAG CG
25.052	HXK1_F	AGG GTT CCA TGG CTG ATG TG
25.053	HXK1_R	ACA TCG TGG CCT TCG ACA TT
25.054	ACT1_F	ATG CAA ACC GCT GCT CAA TC
25.055	ACT1_R	CAA TAC CGG CAG ATT CCA AAC C

Figure S1. Quantitative real time RT-PCR analysis of the expression of some selected genes.

Relative Fold-changes of expression compared to the wild-type control was calculated for the indicated genes based on $2^{-\Delta\Delta Ct}$ analysis from the Ct values of RT q-PCR using the actin gene (*ACT1*) as a housekeeping reference. Means and standard errors of the mean (error bars) were calculated from two technical and two biological replicates. Primers used for RT q-PCR are listed below. Strains used were HD56-5A as a wild-type and FSO62-7A for the *rho5* deletion.
