# Supplementary material for: The small GTPase Rho5—Yet another player in yeast glucose signaling
Source: PLoS Genet. 2025 Sep 9;21(9):e1011858. doi: 10.1371/journal.pgen.1011858 (PMC12440216; doi:10.1371/journal.pgen.1011858)
Supplement: S3 Fig — Four exemplary tetrads are shown, with colored circles designating different combinations of gene deletions as indicated. Colony sizes for each combination (determined from pixel area and given as percentage from wild type set at 100%) were determined from 29 tetrads and quantified in the columns of the diagram at the right (n = total number of segregants obtained for each genotype. Error bars are indicated for each mutant combination. Three asterisks indicate highly significant differences with p-values below 0.001; n.s. = not significant). Diploids analyzed were from the cross of a strain carrying a reg1 mig1 double deletion (FSO79-8C) with one carrying a snf1 deletion (HOD201-2D). (PDF) [file pgen.1011858.s003.pdf]

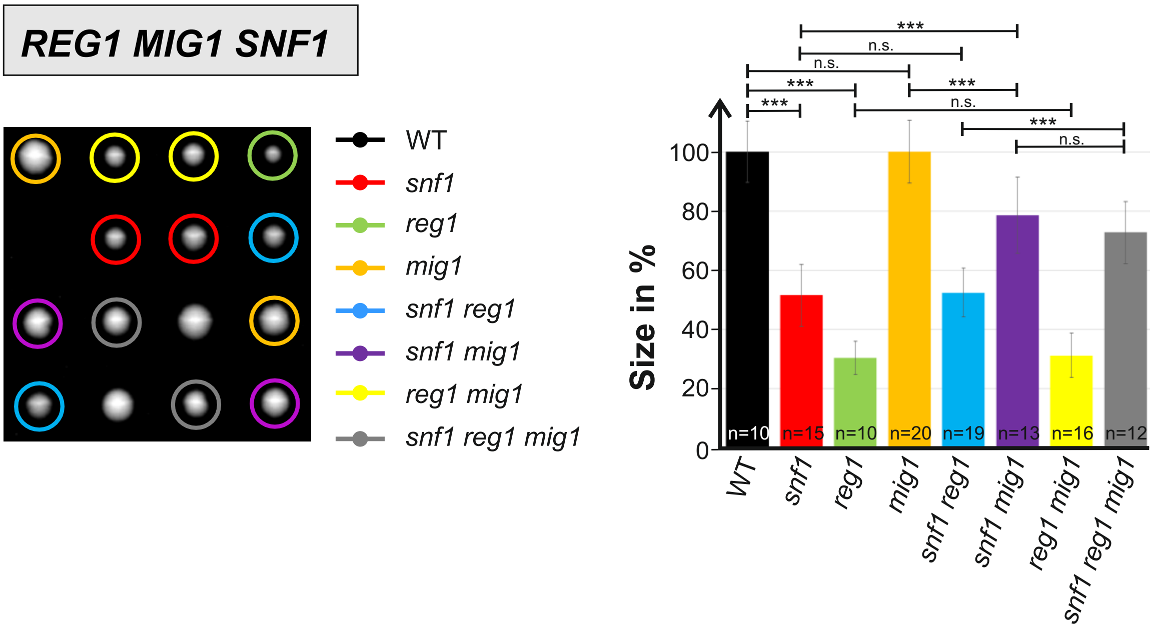

Figure S3. Epistasis analyses based on growth of segregants from tetrad analyses on rich medium (YEPD). Plates four exemplary tetrads are shown, with colored circles designating different combinations of gene deletions as indicated. Colony sizes for each combination (determined from pixel area and given as percentage from wild type set at 100%) were determined from 29 tetrads and quantified in the columns of the diagram at the right (n = total number of segregants obtained for each genotype. Error bars are indicated for each mutant combination. Three asterisks indicate highly significant differences with p-values below 0.001; n.s. = not significant). Diploids analyzed were from the cross of a strain carrying a *reg1 mig1* double deletion (FSO79-8C) with one carrying a *snf1* deletion (HOD201-2D).
