# Supplementary material for: The small GTPase Rho5—Yet another player in yeast glucose signaling
Source: PLoS Genet. 2025 Sep 9;21(9):e1011858. doi: 10.1371/journal.pgen.1011858 (PMC12440216; doi:10.1371/journal.pgen.1011858)
Supplement: S4 Fig — Significance is indicated by one asterisk, while three asterisks indicate a very high significance. Note that differences between each strain with and without hydrogen peroxide are also highly significant but not highlighted with asterisks here for the sake of clarity. (PDF) [file pgen.1011858.s004.pdf]

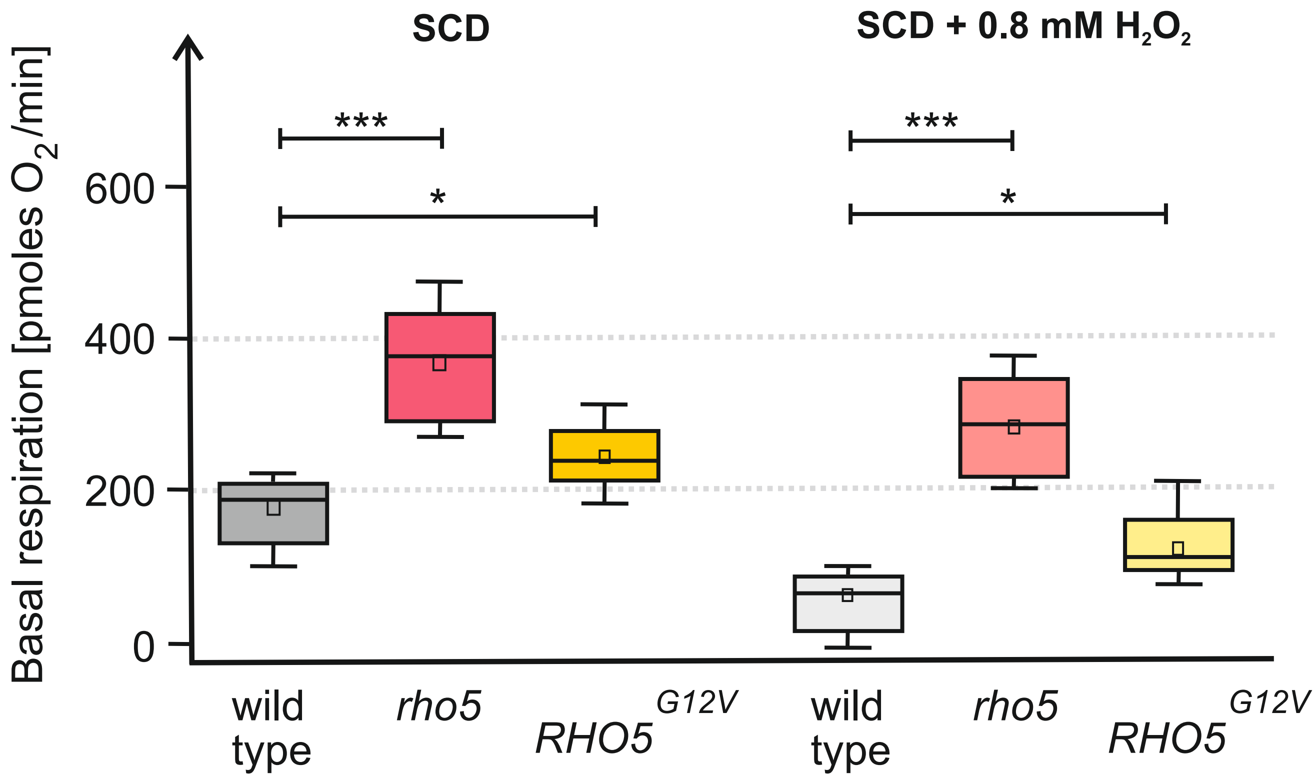

Figure S4. Respiration measurement of a wild type strain (HD56-5A), a *rho5* deletion strain (FSO62-7A) and a *RHO5*^{G12V} mutant strain (HLBO37-4D). Significance is indicated by one asterisk, while three asterisks indicate a very high significance. Note that differences between each strain with and without hydrogen peroxide are also highly significant but not shown here for the sake of clarity.
