# Supplementary material for: The small GTPase Rho5—Yet another player in yeast glucose signaling
Source: PLoS Genet. 2025 Sep 9;21(9):e1011858. doi: 10.1371/journal.pgen.1011858 (PMC12440216; doi:10.1371/journal.pgen.1011858)
Supplement: S5 Fig — Four exemplary tetrads are shown, each, with colored circles designating different combinations of gene deletions as indicated. Colony sizes for each combination (determined from pixel area and given as percentage from wild type set at 100%) were determined and quantified in the columns of the diagram at the right (n = total number of segregants obtained for each genotype. Error bars are indicated for each mutant combination. Three asterisks indicate highly significant differences with p-values below 0.001; n.s. = not significant). Diploids analyzed were: A) From the cross of a strain carrying a reg1 deletion (FSO79-7C) with one carrying a ras2 deletion (HOD320-6A); and B) from the heterozygous diploid strain for reg1 rho5 RAS2G19V transformed with an IME1 expression plasmid (HOD666/IME1). (PDF) [file pgen.1011858.s005.pdf]

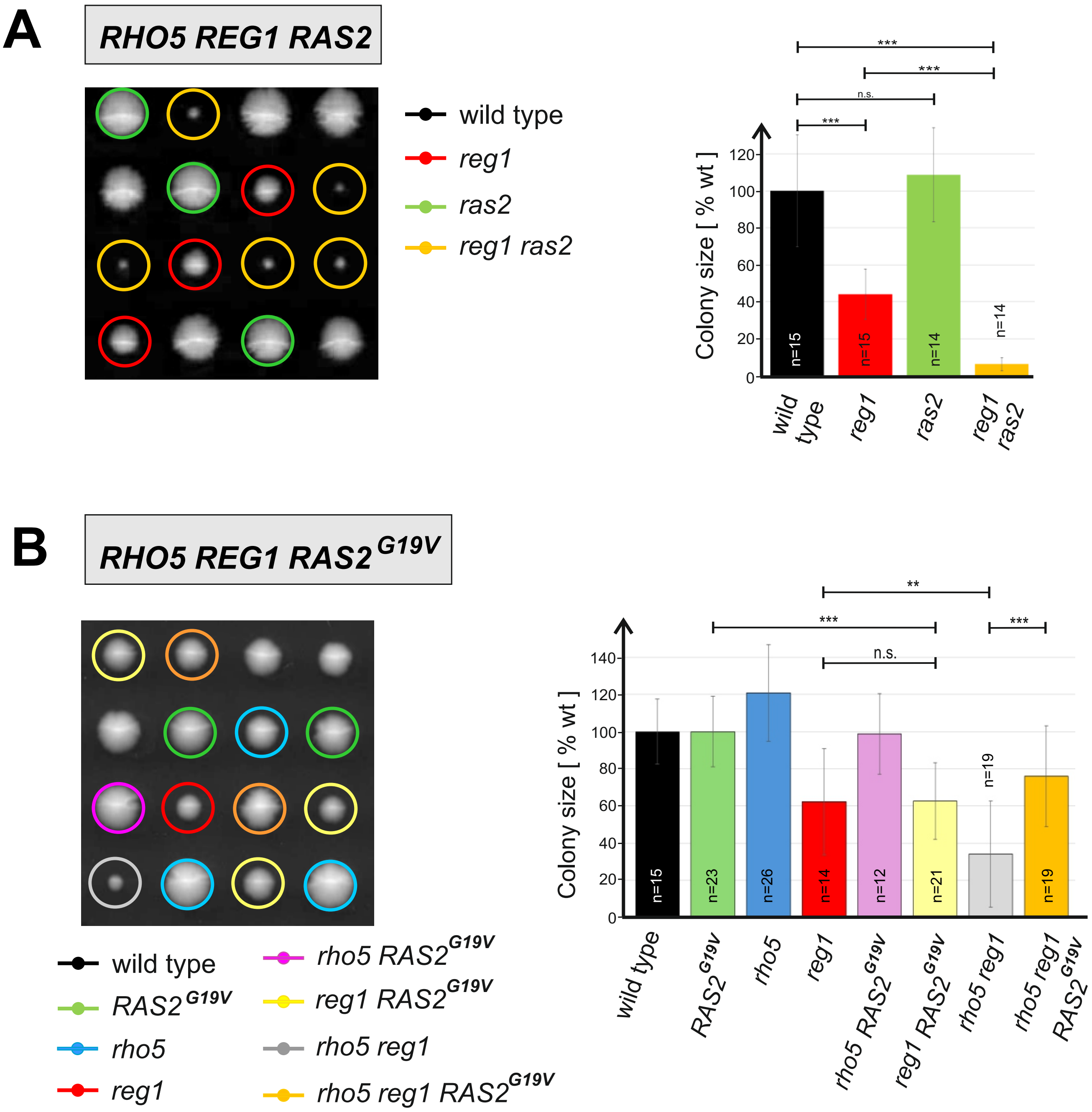

Figure S5. Epistasis analyses for mutants in *REG1, RHO5* and *RAS2* based on growth of segregants from tetrad analyses on rich medium (YEPD). Four exemplary tetrads are shown, each, with colored circles designating different combinations of gene deletions as indicated. Colony sizes for each combination (determined from pixel area and given as percentage from wild type set at 100%) were determined and quantified in the columns of the diagram at the right (n = total number of segregants obtained for each genotype. Error bars are indicated for each mutant combination. Three asterisks indicate highly significant differences with p-values below 0.001; two asterisks with p-values below 0.005; n.s. = not significant). Diploids analyzed were: A) From the cross of a strain carrying a *reg1* deletion (FSO79-7C) with one carrying a *ras2* deletion (HOD320-6A); and B) from diploid strains being heterozygous for *reg1 rho5 RAS2*^{G19V} transformed with an *IME1* expression plasmid (HOD675/IME1 and HOD677/IME1). Note that spores from the latter crosses were allowed to germinate on rich YEPD medium supplemented with adenine and tryptophan.
