# Supplementary material for: The small GTPase Rho5—Yet another player in yeast glucose signaling
Source: PLoS Genet. 2025 Sep 9;21(9):e1011858. doi: 10.1371/journal.pgen.1011858 (PMC12440216; doi:10.1371/journal.pgen.1011858)
Supplement: S6 Fig — Growth curves were recorded on synthetic medium with 2% glucose (SCD), as described in material and methods of the main text, with or without hydrogen peroxide as indicated. Error bars give the standard deviations at each time point obtained from at least two biological and two technical replicates. Genotypes of the otherwise isogenic strains are listed in Table 2. Strains employed were wild type (FSO35-2A and FSO35-4A), RHO5G¹²V, (FSO88-3A and FSO88-2B), yak1 (FSO88-2C and FSO88-6A), RHO5G¹²V yak1 (FSO88-2A and FSO88-1C). (PDF) [file pgen.1011858.s006.pdf]

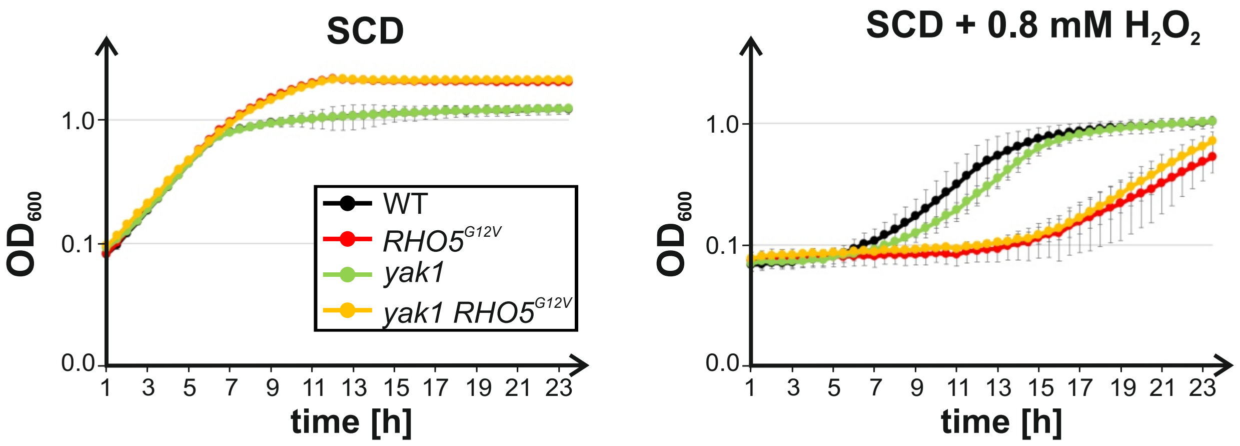

Figure S6. Epistasis analyses of *rho5* and *yak1* mutant combinations based on their sensitivity towards hydrogen peroxide. Growth curves were recorded on synthetic medium with 2% glucose (SCD), as described in materials and methods of the main text, with or without hydrogen peroxide as indicated. Errot bars give the standard deviations at each time point obtained from at least two biological and two technical replicates. Genotypes of the otherwise isogenic strains are listed in Table 2. Strains employed were wild type (FSO35-2A and FSO35-4A), *RHO5*^{G12V}, (FSO88-3A and FSO88-2B), *yak1* (FSO88-2C and FSO88-6A), *RHO5*^{G12V} *yak1* (FSO88-2A and FSO88-1C).
